# Reduced smooth muscle-fibroblasts transformation potentially decreases intestinal wound healing and colitis-associated cancer in ageing mice

**DOI:** 10.1038/s41392-023-01554-w

**Published:** 2023-08-09

**Authors:** Yi Liu, Yanhong Ji, Ruiyi Jiang, Chao Fang, Gang Shi, Lin Cheng, Yinan Zuo, Yixin Ye, Xiaolan Su, Junshu Li, Huiling Wang, Yuan Wang, Yi Lin, Lei Dai, Shuang Zhang, Hongxin Deng

**Affiliations:** 1 Department of Biotherapy, Cancer Center and State Key Laboratory of Biotherapy, West China Hospital, Sichuan University, 610041 Chengdu, Sichuan The People’s Republic of China; 2 Department of Gastrointestinal Surgery, West China Hospital and State Key Laboratory of Biotherapy, Sichuan University, 610041 Chengdu, Sichuan The People’s Republic of China; 3 Respiratory Microbiome Laboratory, Institute of Respiratory Health, Frontiers Science Center for Disease-related Molecular Network, West China Hospital, Sichuan University, 610041 Chengdu, Sichuan The People’s Republic of China; 4 Department of Biotherapy, Cancer Center, West China Hospital, Sichuan University and Collaborative Innovation Center for Biotherapy, 610041 Chengdu, Sichuan The People’s Republic of China

**Keywords:** Gastrointestinal cancer, Cancer microenvironment, Senescence

## Abstract

Cancer and impaired tissue wound healing with ageing are closely related to the quality of life of the elderly population. Given the increased incidence of cancer and the population ageing trend globally, it is very important to explore how ageing impairs tissue wound healing and spontaneous cancer. In a murine model of DSS-induced acute colitis and AOM/DSS-induced colitis-associated cancer (CAC), we found ageing significantly decreases intestinal wound healing and simultaneous CAC initiation, although ageing does not affect the incidence of AOM-induced, sporadic non-inflammatory CRC. Mechanistically, reduced fibroblasts were observed in the colitis microenvironment of ageing mice. Through conditional lineage tracing, an important source of fibroblasts potentially derived from intestinal smooth muscle cells (ISMCs) was identified orchestrating intestinal wound healing and CAC initiation in young mice. However, the number of transformed fibroblasts from ISMCs significantly decreased in ageing mice, accompanied by decreased intestinal wound healing and decreased CAC initiation. ISMCs-fibroblasts transformation in young mice and reduction of this transformation in ageing mice were also confirmed by ex-vivo intestinal muscular layer culture experiments. We further found that activation of YAP/TAZ in ISMCs is required for the transformation of ISMCs into fibroblasts. Meanwhile, the reduction of YAP/TAZ activation in ISMCs during intestinal wound healing was observed in ageing mice. Conditional knockdown of YAP/TAZ in ISMCs of young mice results in reduced fibroblasts in the colitis microenvironment, decreased intestinal wound healing and decreased CAC initiation, similar to the phenotype of ageing mice. In addition, the data from intestine samples derived from inflammatory bowel disease (IBD) patients show that activation of YAP/TAZ also occurs in ISMCs from these patients. Collectively, our work reveals an important role of the ageing stromal microenvironment in intestinal wound healing and CAC initiation. Furthermore, our work also identified a potential source of fibroblasts involved in colitis and CAC.

## Introduction

Although colorectal cancer (CRC) is usually regarded as an age-related disease because of accumulated mutations with ageing, the relationship between the ageing stromal microenvironment and CRC is poorly understood. CRC is a typical cancer associated with tissue inflammation. The increased incidence of CRC is also associated with factors such as environmental exposures, lifestyle (e.g., smoking, alcohol consumption, eating red or processed meat), and disease conditions (e.g., obesity, diabetes, and inflammatory bowel disease).^1^ These factors could promote cancer by acting in whole or in part via inflammation.^2^ Compared to the certain role of accumulated mutations with ageing, it is still unclear as to whether and how the ageing stromal microenvironment could impact CRC, especially in inflammation-driven CRC.

Inflammation immensely remodels the tissue microenvironment and plays an important role in nearly all stages of tumorigenesis.^3^ Mechanistically, in addition to the important role of genomic instability and accumulated mutagenesis in the inflammatory microenvironment to promote carcinogenesis, infiltrated and resident cells also play important roles in cancer formation, including fibroblasts, macrophages, neutrophils and others.^4,5^ Besides, as responses to tissue damage and inflammation, re-epithelialization and tissue regeneration during wound healing also play important roles in cancer formation.^6,7^ For example, it was revealed that skin wound healing promotes skin carcinogenesis^8,9^, and the wound healing response to surgery promotes the outgrowth of dormant breast tumor cells.^10^

Meanwhile, ageing is a complex process associated with the degeneration and functional decline of nearly all organs and body systems.^11^ The degeneration of wound healing and tissue regeneration with ageing are also observed in some tissues, mainly including the liver, skin, small intestine and muscles.^12–15^ Therefore, it warrants further exploration as to whether impaired wound healing with ageing would negatively affect carcinogenesis under inflammatory conditions. Although some studies observed that ageing could impair the severity of colitis,^16–18^ it is still unclear whether and how ageing would impact intestinal wound healing and inflammation-driven CRC in the colon.

In this report, we mainly used a murine model of AOM/DSS-induced colitis-associated cancer (CAC) to investigate the relationship between ageing and inflammation-driven CRC. The CAC in AOM/DSS-induced mice usually occurs in the distal part of the colon, which resembles the location of CRC at the distal colon and rectum in human.^19^ Our work reveals that reduced fibroblasts potentially derived from ISMCs in the ageing intestinal stromal microenvironment could significantly decrease wound healing following DSS-induced tissue damage and simultaneously decrease the initiation of CAC. As the crucial regulator of cell plasticity and cell fate determination,^20^ transcriptional co-activators YAP/TAZ activation are found to be required for the transformation of ISMCs into fibroblasts during intestinal wound healing in murine CAC and acute colitis models in our study. Taken together, our study indicates that an ageing intestinal stromal microenvironment could significantly impair intestinal wound healing and inflammation-driven CRC. Our study also reveals a new mechanism for intestinal wound healing in young mice and uncovers the important role of YAP/TAZ in intestinal smooth muscle cells.

## Results

### Ageing does not affect colon homeostasis under non-inflammatory conditions

To explore whether the ageing process (middle-aged stage) affects colon homeostasis under non-inflammatory conditions, we assessed the differences in homeostatic colon between young (~3 months) and ageing (~14 months, corresponding to nearly 50 years in humans) mice.^21^ We observed no obvious differences in colon length and tissue lesions between the young and ageing mice (Supplementary Fig. 1a, b). Tissue structure of both young and ageing colons was characterized by stratified structures mainly composed of intestinal mucosa epithelium (IE), muscularis mucosae (mm) and muscular layer (ml) (Supplementary Fig. 1c). Both the ratio of proliferating cells and Lgr5^+^ colon stem cells in intestinal crypts of the colon were comparable between young and ageing mice (Supplementary Fig. 1d, e). RNA sequencing (RNA-seq) was further performed to analyze the overall expression profiles of colon tissues derived from young and ageing mice in homeostasis. Comparative expression analysis of duplicates of RNA-seq data revealed that only 75 coding mRNAs were differentially expressed by ±2-fold (*P* < 0.05), and the expression profiles of most differentially expressed genes were modestly changed (less than 4-fold) (Supplementary Fig. 1f). Furthermore, similar intestinal organoids cultured from the same number of young and ageing intestinal crypts of the colon were observed (Supplementary Fig. 1g). Meanwhile, the ageing organoids share the same potency to passage up to at least three generations as young organoids (Supplementary Fig. 1h). These results indicated that the ageing (middle-aged stage) colon maintains a tissue structure that is architecturally and transcriptionally similar to that of young mice under homeostatic conditions.

### Ageing decreases intestinal wound healing in murine acute colitis

**Fig. 1:**
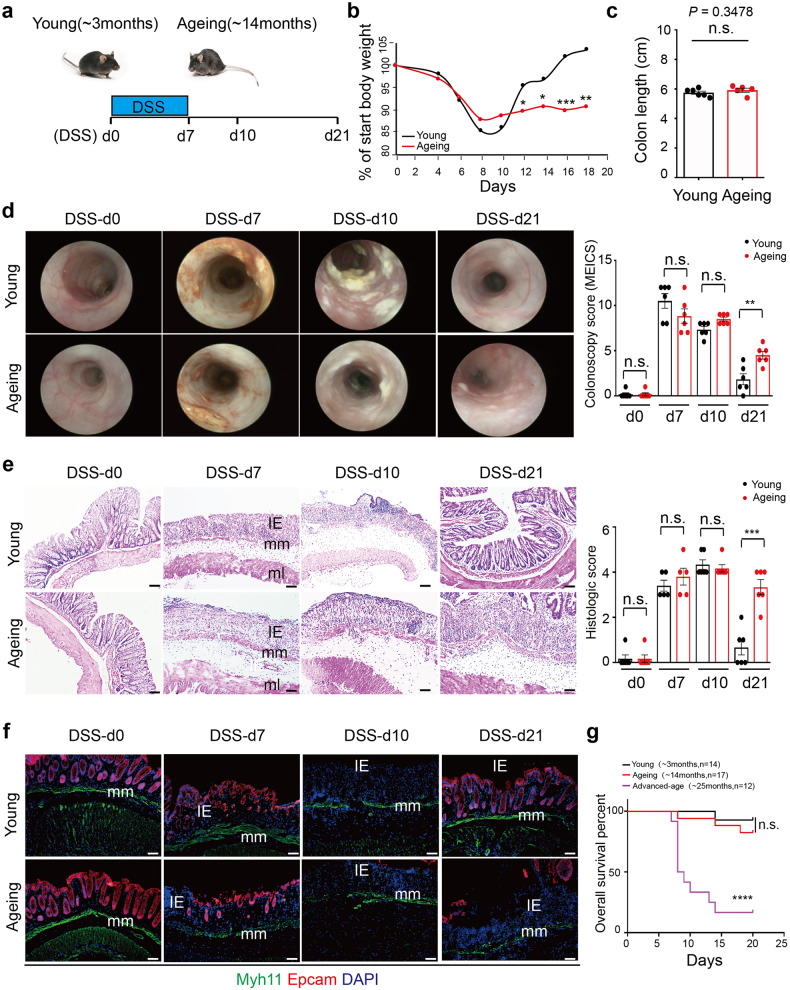
Decreased intestinal wound healing with ageing in DSS-induced acute colitis. **a** Schematic overview of DSS-induced acute colitis. Colon tissues were collected at the early phase of colitis at DSS-d7, during colitis at DSS-d10, and after the wound healing at DSS-d21. **b** The weight change in young and ageing mice during DSS-induced acute colitis. Young, *n* = 12; Ageing, *n* = 9. **c** The colon length of young (*n* = 6) and ageing (*n* = 5) mice at DSS-d7, mean ± SD. **d** High-resolution endoscopic images of young and ageing colon at 0, 7, 10 and 21 days after the beginning of DSS feeding. The severity of colitis was measured using the coloscopy score system (MEICS). *n* = 6 for each group, mean ± SD. **e** Representative H&E staining of colon tissues at 0, 7, 10 and 21 days after the beginning of DSS feeding. IE intestinal mucosa epithelium; mm muscularis mucosa; ml muscular layer; these abbreviations apply to the whole study. The histopathological grading of inflammation in young and ageing mice was recorded. DSS-d0, *n* = 6; DSS-d7, *n* = 5; DSS-d10, *n* = 6; DSS-d21, *n* = 6. Data are mean ± SD. Scale bars, 50 μm. **f** Immunofluorescence images of the temporal re-epithelialization process that occurs following the DSS-induced colitis in young and ageing mice. Sections were immunolabeled with Epcam (marker of epithelium cells) and Myh11 (marker of smooth muscle cells). Scale bars, 50 μm. **g** Survival analysis of the advanced-age (~25 months) mice (purple solid line; *n* = 12) compared to that of young mice (black solid line; *n* = 14; *P* < 0.0001 by log-rank test) during DSS-induced acute colitis. Note that survival of young and ageing (~14 months, red dashed line, *n* = 17) mice had no significant difference

To further explore whether ageing affects intestinal wound healing, we assessed whether ageing affects the inflammation process in DSS-induced murine acute colitis. As the model diagram shows, continuous administration of DSS for up to 7 days (from DSS-d0 to DSS-d7) typically induces tissue damage in the colonic mucosa, accompanied by severe intestinal inflammation (DSS-d7 to DSS-d10) and subsequent intestinal wound healing usually completed until to nearly DSS-d21 (Fig. 1a). With the administration of DSS, although the body weight of both young and ageing mice began to drop and reached the comparable ratio of lost weight 10 days after the beginning of DSS administration (DSS-d10). Yet, the body weight of young, but not ageing mice, recovered to the normal level quickly subsequently (Fig. 1b). After DSS-induced tissue damage, the colon length was similar between young and ageing mice at DSS-d7 (Fig. 1c). Using the in vivo coloscopy system, we observed that ageing mice acquired the same score of endoscopic colitis upon DSS-induced damage as young mice at DSS-d7 and DSS-d10, but subsequently the colitis in ageing mice failed to recover at DSS-d21 (Fig. 1d). Similarly, disorganized tissue structure and the loss of epithelial marker EPCAM after DSS-induced tissue damage were observed at DSS-d7 and DSS-d10 in both young and ageing mice, but only the young mice showed quick re-epithelization after intestinal wound healing at DSS-d21 (Fig. 1e, f). To further assess the potential effect of ageing on intestinal wound healing, the mice at the age of ~25 months (advanced-age group) were used to induce colitis. To our surprise, nearly half of the advanced-age mice failed to tolerate the DSS-induced tissue damage, and the rest of the advanced-age mice failed to recover and ultimately died of colitis (Fig. 1g). Taken together, our results indicate that ageing significantly decreases intestinal wound healing in DSS-induced acute colitis.

### Ageing decreases the initiation of colitis-associated cancer (CAC)

**Fig. 2:**
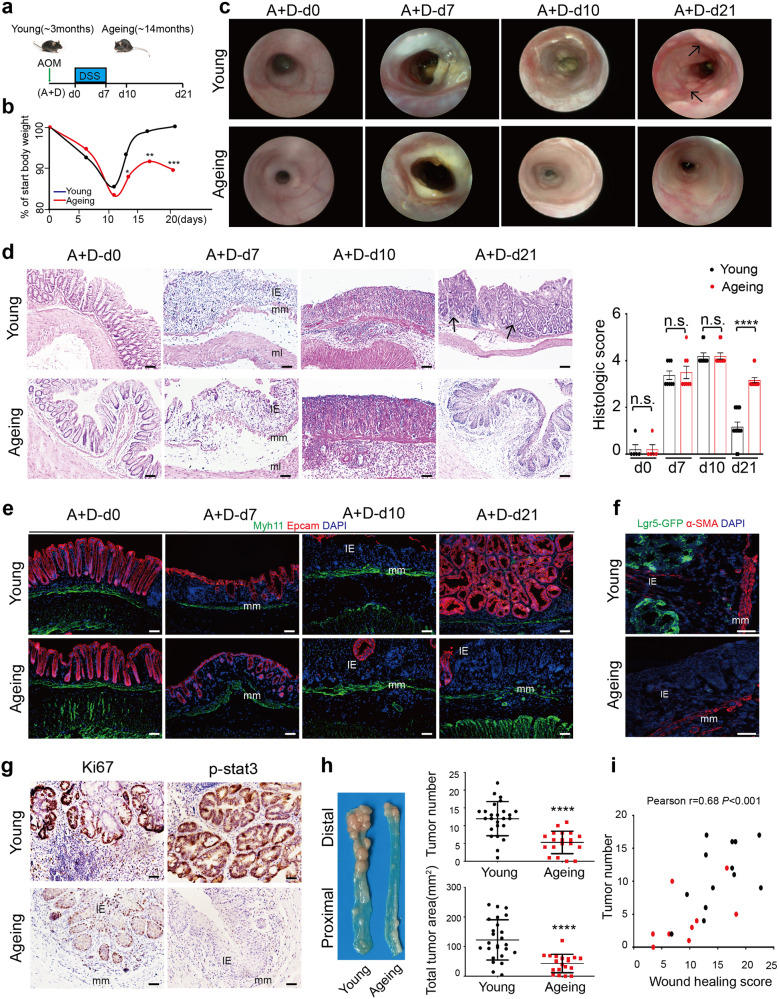
Decreased initiation of colitis-associated cancer in ageing mice. **a** Schematic overview of the early phase of the CAC model, A+D-d0 means normal mice. **b** Weight change during the early phase of CAC in young (~3 months) and ageing (~14 months) mice. Young, *n* = 15; Ageing, *n* = 11. **c** High-resolution endoscopic images of young and ageing colon at 0, 7, 10 and 21 days after the beginning of DSS feeding. **d** Representative H&E staining of colon tissues at 0, 7, 10 and 21 days after the beginning of DSS feeding. The histopathological grading of inflammation in young and ageing mice was recorded. A+D-d0, *n* = 5; A+D-d7, *n* = 8; A+D-d10, *n* = 10; A+D-d21, *n* = 12. Data are mean ± SD. Scale bars, 50 μm. **e** Immunofluorescence images of the temporal re-epithelialization process during the early phase of the CAC model in young and ageing mice. Sections were immunolabeled with Epcam and Myh11. Scale bars, 50 μm. **f** Detection of GFP and α-SMA at A+D-d21 in colon tissues of young and ageing Lgr5-eGFP^+/-^ mice. Scale bars, 50 μm. **g** Immunohistochemical staining of Ki67 and p-stat3 (malignant transformation marker) in colon tissues at A+D-d21 in young and ageing mice. Scale bars, 50 μm. **h** Representative macroscopic images of CAC in young and ageing mice at A+D-d63. Tumor number and tumor area per colon were measured. Young, *n* = 25; Ageing, *n* = 19, *P* < 0.0001. Data are mean ± SD. **i** Pearson correlation between the wound healing score during the early phase of the CAC model and the parallel tumor numbers at A+D-d63. The wound healing score was measured by the ratio of recovered body weight to the start body weight. Black spot represents one young mouse, and red spot represents one ageing mouse. Pearson r = 0.68, *P* < 0.001, *n* = 22

Given the important role of tissue regeneration in cancer formation,^6,7^ we continued to explore whether the decreased intestinal wound healing in ageing mice influences the initiation of murine CAC, which is usually composed of one injection of AOM to accumulate the necessary mutations and subsequent three cycles of DSS-induced acute colitis (Supplementary Fig. 2a). After tissue regeneration during intestinal wound healing of the first cycle of DSS-induced colitis, CAC initiation usually occurred at A+D-d21, accompanied with raised macroscopic polyps and precancerous lesions.^19,22^ Therefore, we recognize the first cycle of colitis as the early phage of CAC and collected the distal part of the colon at days 0 (A+D-d0), 7 (A+D-d7), 10 (A+D-d10), 14(A+D-d14), 21 (A+D-d21) upon DSS administration respectively (Fig. 2a). Consistent with what was observed in DSS-induced acute colitis, the ageing mice showed the same severity of DSS-induced tissue damage at A+D-d7 and A+D-d10, but exhibited the reduction in weight recovery, organizational reconstruction, as well as re-epithelization during the wound healing of the early phase of CAC, at A+D-d14 and A+D-d21 (Fig. 2b–e and Supplementary Fig. 2c–e). Simultaneously, through the in vivo coloscopy system, raised macroscopic polyps were observed at A+D-d21 only in the young mice but not in ageing mice (Fig. 2c). Besides, the typical precancerous tissue structure was observed only in young mice at A+D-d21, but not in ageing mice (Fig. 2d, e). Compared to typical crypt and precancerous structure in young mice of the distal colon, typical and large-scale squamous epithelium were observed in ageing mice at A+D-d21 (Supplementary Fig. 2e, f). Given the discovery about transitional anal squamous epithelium in colitis repair from D. Brent Polk,^23^ we speculate that decreased wound healing and re-epithelization in ageing colon need help from anal squamous epithelium to build more squamous epithelium barrier necessarily. We further observed reduced colon stem cells (lgr5^+^), reduced proliferation (ki67^+^) of epithelial cells and reduced precancerous lesions (p-stat3^+^) of epithelial cells in ageing colon tissues, compared to those in young colon tissues at A+D-d21 (Fig. 2f, g and Supplementary Fig. 2b). When the CAC model was completed (A+D-d63), the total tumor number and tumor area in the distal part of the colon were decreased in ageing mice compared with that in young mice (Fig. 2h and Supplementary Fig. 2g, h). In addition, for each mouse, a positive correlation between tumor number at A+D-d63 and the parallel wound healing score (assessed by the ratio of recovered body weight/start body weight) during the early phase of CAC was observed in our study (Fig. 2i). Altogether, these results indicate that ageing process significantly decreases intestinal wound healing and simultaneously decreases CAC initiation.

To further explore the mechanism underlying decreased CAC initiation and formation in ageing mice, we used multiple injections of AOM-induced sporadic non-inflammatory CRC to explore whether ageing impairs carcinogenesis of AOM-induced mutations in intestinal epithelial cells (As model diagram shown, Supplementary Fig. 3a). One week after the first injection of AOM, comparative expression analysis of duplicates of RNA-seq data revealed that only 86 coding mRNAs were differentially expressed by ±2-fold (*P* < 0.05) between the young and ageing colon (Supplementary Fig. 3b). Using an in vivo coloscopy system, we observed that tumor initiation and development were almost synchronous in young and ageing mice (Supplementary Fig. 3c). As shown (Supplementary Fig. 3d–g), 25 weeks after the first time of AOM injection, tumor number, tumor area, the pathological structure of tumors, PCNA and p-stat3 in tumor cells were comparable between young and ageing mice. The phenotypical characterization indicates that ageing has no obvious effect on the formation of AOM-induced sporadic CRC under non-inflammatory conditions.

### Reduced fibroblasts with ageing in colitis microenvironment of murine acute colitis and the early phase of CAC

As the first step toward understanding the mechanism underlying impaired wound healing in the early phase of CAC in ageing mice, we found both the ratio of total immune cells and the ratio of main subtypes of immune cells in the intestinal microenvironment after DSS-induced tissue damage were similar between young and ageing mice (Supplementary Fig. 4a–c). In addition, the elevated inflammatory factors in response to DSS-induced tissue damage were similar in the colon tissues of young and ageing mice (Supplementary Fig. 4d). Therefore, these data suggest that the number of immune cells present after DSS-induced tissue damage seems similar between young and ageing mice, accompanied with a similar phenotype of the severity of tissue damage described above.

**Fig. 3:**
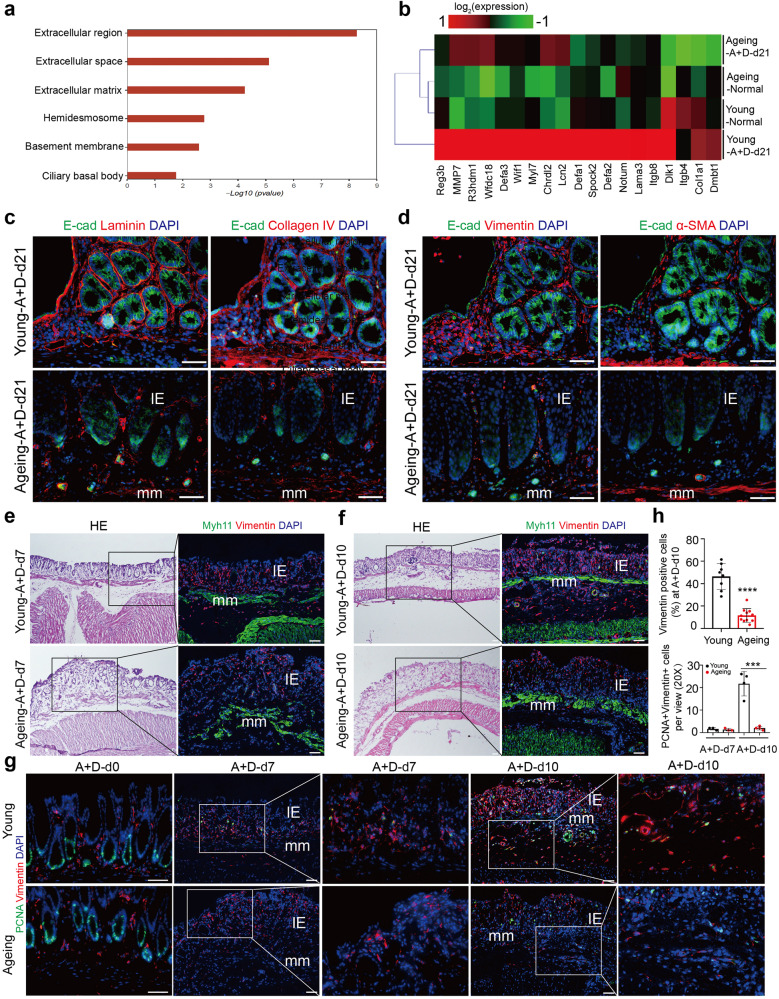
Reduced fibroblasts with ageing during intestinal wound healing in the early phase of CAC. **a** Enriched gene ontology (GO) terms in genes that were differentially regulated between young and ageing colon tissues at A+D-d21. *n* = 3 for each group. **b** Heatmap of differentially regulated mRNAs associated with extracellular matrix between young and ageing colon tissues at A+D-d21. *n* = 3 for each group. **c** Detection of Laminin, Collagen IV and E-cad at A+D-d21 in young and ageing mice. Scale bars, 50 μm. **d** Detection of Vimentin, E-cad and α-SMA at A+D-d21 in young and ageing mice. Scale bars, 50 μm. **e** Detection of Vimentin and Myh11 at A+D-d7 in young and ageing mice. Scale bars, 50 μm. **f** Detection of Vimentin and Myh11 at A+D-d10 in young and ageing mice. Scale bars, 50 μm. **g** Detection of Vimentin and PCNA in colon tissues of young and ageing mice at A+D-d0, A+D-d7 and A+D-d10. Scale bars, 50 μm. **h** The ratio of Vimentin^+^ fibroblasts in young and ageing intestinal stromal microenvironment at A+D-d10 was detected, Young, *n* = 8; Ageing, *n* = 11; Data are mean ± SD. The absolute number of PCNA^+^ Vimentin^+^ cells per view (20X) was also detected at A+D-d7 and A+D-d10, Young, *n* = 3; Ageing, *n* = 4; Data are mean ± SD

To further explore the mechanism, RNA-sequencing and gene ontology (GO) analysis were performed using the colon samples obtained from young and ageing mice at A+D-d21. Gene ontology (GO) analysis illustrated that some differential expression of mRNAs between young and ageing colon tissues at A+D-d21 were specifically enriched in the extracellular region and matrix, which were decreasingly expressed in the ageing intestinal microenvironment (Fig. 3a, b). The dysregulated extracellular matrix was further confirmed at the protein level using immunofluorescence staining of Laminin and Collagen-IV in ageing mice (Fig. 3c).

Given the important roles of fibroblasts in wound healing and cancer formation through regulating the extracellular matrix, tissue structure, stem cell maintenance and self-renewal, and others,^4,24^ we postulated that the dysregulated extracellular matrix and decreased wound healing in ageing mice might be associated with fibroblasts. Vimentin is regarded as the bottommost marker of fibroblasts, and alpha-smooth muscle actin (α-SMA) is usually used to identify activated fibroblasts (myofibroblasts) which are also usually regarded as cancer-associated fibroblasts (CAFs) in the tumor microenvironment.^25^ Indeed, the reduced fibroblasts and myofibroblasts in the ageing intestinal microenvironment at A+D-d21 were observed (Fig. 3d). Of note, we did not observe significant differences in resident fibroblasts and extracellular matrix of the colon between young and ageing mice under homeostatic conditions (Supplementary Fig. 5a, b).

To further explore fibroblasts in the colitis microenvironment during intestinal wound healing of the early phase of the CAC model, we dynamically detected the fibroblasts at A+D-d7 and A+D-d10 in young and ageing mice. After DSS administration (A+D-d7), sporadic fibroblasts were detected in intestinal mucosa epithelium in both young and ageing mice (Fig. 3e). Yet, as colitis progressed, abundant fibroblasts were observed in the intestinal microenvironment of young mice at A+D-d10, including a significant population of fibroblasts around the muscularis mucosa, but not observed in ageing mice (Fig. 3f–h). Meanwhile, we found that the resident fibroblasts under homeostatic conditions and in the early phase of colitis (A+D-d7) rarely proliferate, as indicated by PCNA-negative staining in both young and ageing mice (Fig. 3g). However, the fibroblasts detected at A+D-d10, especially those around the muscularis mucosa began to proliferate only in young mice (Fig. 3g, h). Similar to what was observed in the early phase of the CAC model, the reduced fibroblasts during intestinal wound healing (DSS-d10) were also observed in DSS-induced acute colitis in ageing and advanced-age mice (Supplementary Fig. 5c–e). All these data indicate that there are reduced fibroblasts during intestinal wound healing in ageing mice, accompanied by a decrease in both intestinal wound healing and CAC initiation.

### Intestinal smooth muscle cells (ISMCs) potentially transform into fibroblasts orchestrating intestinal wound healing and CAC only in young mice

**Fig. 4:**
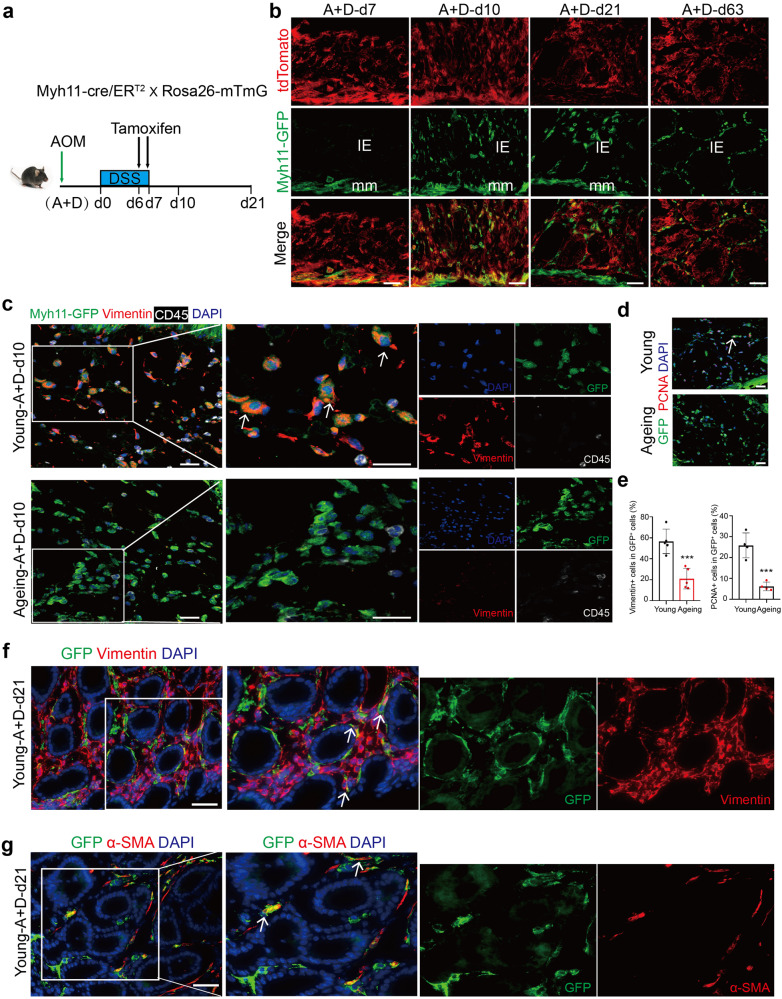
ISMCs transform into fibroblasts orchestrating intestinal wound healing and CAC in young mice. **a** Schematic overview of the tamoxifen induction to label ISMCs during the early phase of the CAC model. **b** After the two continuous days of tamoxifen (3 mg/day) induction starting from A+D-d6, we detected the labeled cells derived from ISMCs (Green) and the total cells (tdTomato, red) in colon tissues at A+D-d7, A+D-d10, A+D-d21 and A+D-d63. Scale bars, 50 μm. **c** Detection of the GFP, Vimentin and CD45 around muscularis mucosa at A+D-d10 in young and ageing Myh11-cre/ER^T2^; Rosa26-mTmG mice. Scale bars, 20 μm. **d** Detection of the GFP and PCNA around muscularis mucosa at A+D-d10 in young and ageing Myh11-cre/ER^T2^; Rosa26-mTmG mice. Scale bars, 20 μm. **e** The ratio of Vimentin^+^ cells in GFP^+^ labeled cells around muscularis mucosa at A+D-d10 in young and ageing Myh11-cre/ER^T2^; Rosa26-mTmG mice. Young, *n* = 5; Ageing, *n* = 5; Data are mean ± SD. At the same time, the ratio of PCNA^+^ cells in GFP^+^ labeled cells around muscularis mucosa was also detected, *n* = 4. Data are mean ± SD. **f** Detection of the GFP and Vimentin in the early stage of CAC at A+D-d21 in young Myh11-cre/ER^T2^; Rosa26-mTmG mice. Scale bars, 50 μm. **g** Detection of the GFP and α-SMA in the early stage of CAC at A+D-d21 in young Myh11-cre/ER^T2^; Rosa26-mTmG mice. Scale bars, 50 μm

Given the occurrence of the proliferating fibroblasts around muscularis mucosa during intestinal wound healing in young mice, we hypothesized that ISMCs might be a potential source of fibroblasts during intestinal wound healing. To test this hypothesis, we utilized young Myh11-cre/ER^T2^; mTmG lineage tracing system to conditionally label ISMCs, allowing it to convert resident red fluorescent protein (tdTomato) into green fluorescent protein (GFP) upon treatment with tamoxifen. To explore the best time to specially label smooth muscle cells, we first detected the expression of smooth muscle marker (Myh11) in colon tissues. Under the homeostatic condition, in addition to muscularis mucosae (mm) and muscular layer (ml), Myh11 was also detected in stromal cells in intestinal mucosa epithelium (Supplementary Fig. 6b), which may be expressed on the tissue-resident myofibroblasts.^26^ As a result, a small number of tissue-resident cells in the intestinal mucosa epithelium could be labeled with GFP after tamoxifen induction starting from homeostatic condition (Supplementary Fig. 6a), which brings us difficulty in answering whether the subsequently observed GFP^+^ labeled cells in epithelium mucosae at DSS-d10 come from tissue-resident myofibroblasts or smooth muscle cells. However, the expression of Myh11 was nearly lost in intestinal resident myofibroblasts encountered with DSS-induced tissue damage at A+D-d7 and A+D-d10 (Supplementary Fig. 6b), which is considered the best time frame to label ISMCs specifically. Therefore, we injected tamoxifen to label ISMCs at day 6 and day 7 of the DSS feeding week, respectively, in the early phase of CAC (Fig. 4a). Through this lineage-tracing strategy, in addition to ISMCs, almost no GFP^+^ labeled cells were detected in intestinal mucosa epithelium 36 hours after the beginning of tamoxifen induction at A+D-d7 (Fig. 4b). However, with the progression of colitis, a significant population of GFP^+^ labeled cells potentially derived from smooth muscle were observed in the intestinal mucosa epithelium at A+D-d10, which were present persistently throughout wound healing at the early stage of CAC (A+D-d21) and even in CAC (A+D-d63, Fig. 4b).

With the same lineage-tracing strategy in young mice (Fig. 4a), a population of GFP^+^ labeled cells was also observed in the intestinal microenvironment of ageing Myh11-cre/ER^T2^; mTmG mice at A+D-d10 and these cells lost smooth muscle cell marker α-SMA which were similar to that in young mice (Supplementary Fig. 6c, d). However, nearly 60% of the labeled cells around the muscularis mucosa and labeled cells in the intestinal mucosa epithelium became Vimentin^+^ fibroblasts only in young mice but not in ageing mice (Fig. 4c–e and Supplementary Fig. 6e). Besides, proliferating labeled cells around the muscularis mucosa were observed only in young mice at A+D-d10, but not in ageing mice (Fig. 4d, e). Meanwhile, the labeled Vimentin^+^ fibroblasts and α-SMA^+^ activated fibroblasts were also observed in precancerous tissue at A+D-d21 in young mice (Fig. 4f, g). These data suggest that the fibroblasts newly transformed from ISMCs might be a potential source of fibroblasts involved in intestinal wound healing and CAC initiation seen in young mice. However, this transformation was significantly reduced during intestinal wound healing in ageing mice.

**Fig. 5:**
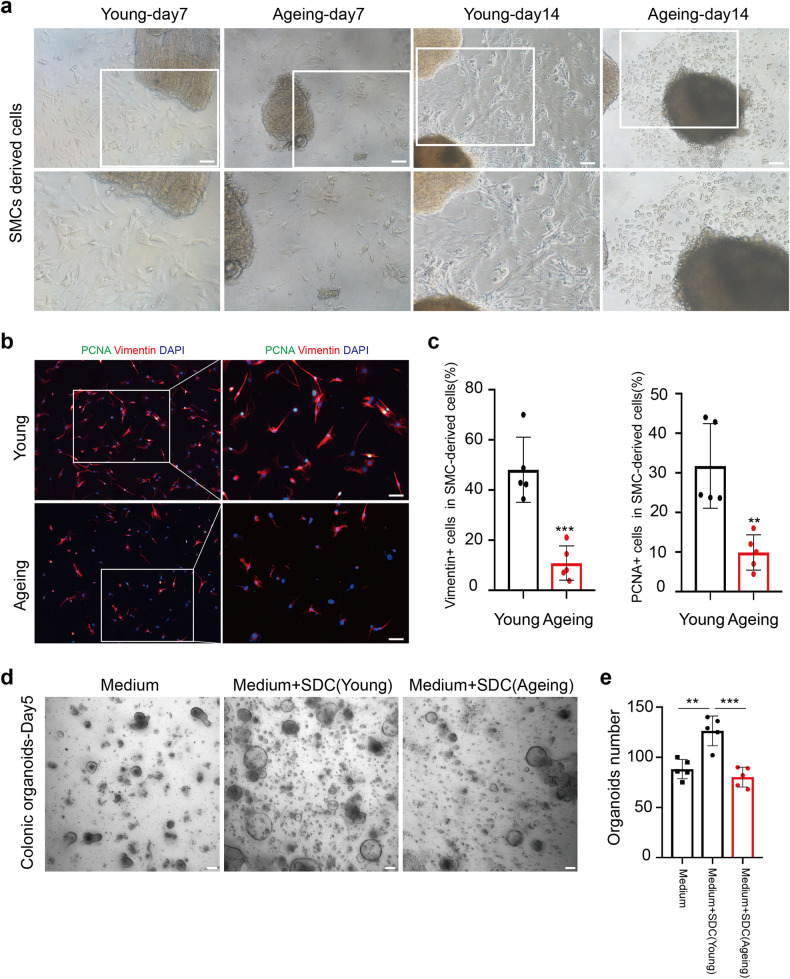
Ageing reduces fibroblasts derived from the ex-vivo culture of the muscular layer. **a** SMCs derived cells (SDCs) from the muscular layer of young and ageing mice at day 7 and day 14. Scale bars, 100 μm. **b** Detection of Vimentin, PCNA and DAPI of SDCs from the muscular layer of young and ageing mice at day 14. Scale bars, 50 μm. **c** The ratio of Vimentin^+^ cells and PCNA^+^ cells in SDCs from the young and ageing muscular layer. *n* = 5 for each group, Data are mean ± SD. **d**, **e** Intestinal organoids derived from 400 crypts cultured alone or 400 crypts cocultured with 10000 SDCs from young or ageing mice, organoids number was measured at day 5, *n* = 5 for each group, mean ± SD. Scale bars, 100 μm

To further confirm the transformation of ISMCs into fibroblasts in ex-vivo experiments, we isolate and culture colonic muscle layer tissue from young and ageing mice. By nearly day 7 and day 14, we can observe proliferating fibroblasts-like cells around muscle layer tissue only in young mice, but not the typical non-fibroblasts-like cells in ageing mice, which is identified by Vimentin and PCNA IF staining (Fig. 5a–c). Then we collected emigrated cells derived from smooth muscle (SDCs) of young and ageing mice to coculture with colonic crypts. More colonic organoids were observed in crypts coculture with SDCs from young mice, compared to the same number of colonic crypts cultured alone or cocultured with SDCs from ageing mice (Fig. 5d, e). Similar to the well-known and crucial role of fibroblasts in tissue stem cell maintaining and self-renewal,^24,27–29^ these data indicate that the fibroblasts derived from ISMCs could also take effect on colonic organoids growth.

### Ageing reduces activation of YAP/TAZ in ISMCs during intestinal wound healing

**Fig. 6:**
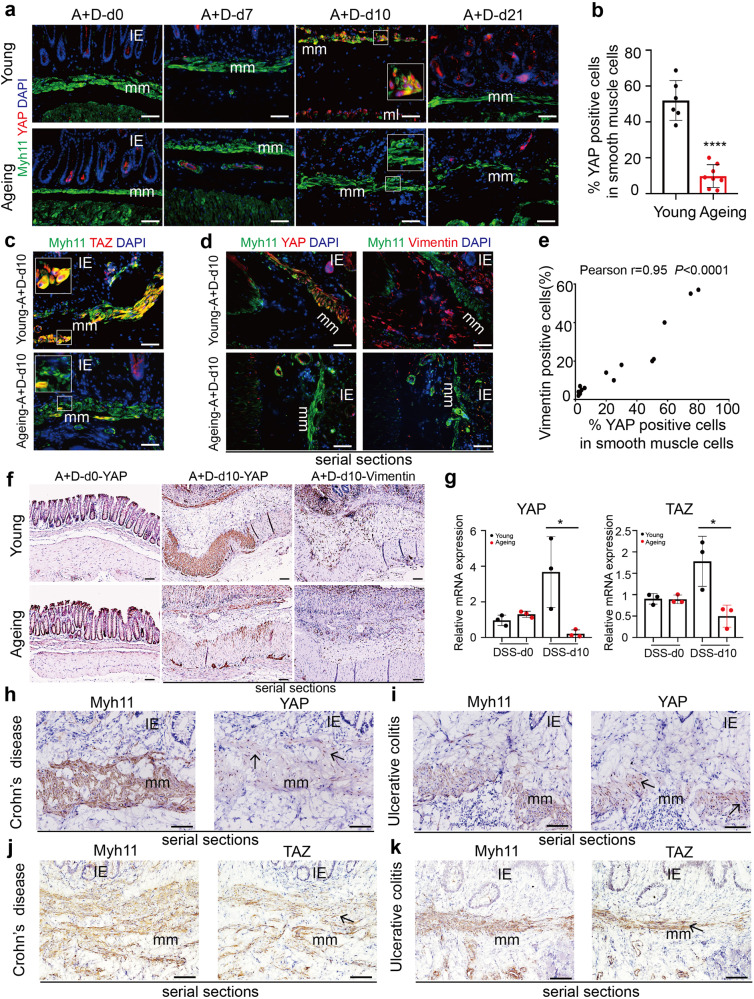
Detection of YAP/TAZ in ISMCs during murine intestinal wound healing and in IBD patients. **a** Detection of the Myh11 and YAP in ISMCs in homeostasis or during the early phase of CAC model in young and ageing mice. Scale bars, 50 μm. **b** The ratio of YAP-positive cells in muscularis mucosa at A+D-d10 in young and ageing mice. Young, *n* = 6; Ageing, *n* = 8; Data are mean ± SD. **c** Detection of the Myh11 and TAZ in ISMCs at A+D-d10 in young and ageing mice. Scale bars, 50 μm. **d** Serial sections detection of YAP, Vimentin and Myh11 around muscularis mucosa at A+D-d10 in young and ageing mice. Scale bars, 50 μm. **e** Pearson’s correlation between the ratio of YAP-positive cells in muscularis mucosa and the ratio of Vimentin^+^ fibroblasts in stromal cells around the muscularis mucosa at A+D-d10. Pearson r = 0.95, *P* < 0.0001, *n* = 15. **f** Immunohistochemical staining of YAP and Vimentin at A+D-d0 and A+D-d10. Scale bars, 50 μm. **g** Relative mRNA expression of YAP and TAZ in the muscular layer of young and ageing mice at DSS-d0 and DSS-d10, *n* = 3 for each group, Data are mean ± SD. **h** Serial sections immunohistochemical staining of Myh11 and YAP in Crohn’s disease patients. Scale bars, 50 μm. **i** Serial sections immunohistochemical staining of Myh11 and YAP in ulcerative colitis patients. Scale bars, 50 μm. **j** Serial sections immunohistochemical staining of Myh11 and TAZ in Crohn’s disease patients. Scale bars, 50 μm. **k** Serial sections immunohistochemical staining of Myh11 and TAZ in ulcerative colitis patients. Scale bars, 50 μm

Given the critical role of transcriptional co-activators YAP/TAZ in cell fate determination,^30^ we speculated that the YAP/TAZ might play an important role in the transformation of ISMCs into fibroblasts. Therefore, we dynamically detected the expression of YAP/TAZ during the early phase of CAC in young and ageing mice. Compared to its low expression in ISMCs under homeostatic conditions, activation of YAP/TAZ and nuclear location in ISMCs during intestinal wound healing at A+D-d10 were evident in young mice but not in ageing mice (Fig. 6a–c). At the same time, the ratio of YAP-positive smooth muscle cells in total ISMCs was positively correlated with the number of fibroblasts around ISMCs at A+D-d10 (Fig. 6d, e). Activation of YAP/TAZ in the young colonic muscle layer at A+D-d10 was also confirmed by IHC and Q-PCR assays (Fig. 6f, g and Supplementary Fig. 7a). After the wound healing, expression of YAP in ISMCs returned to the normal level at A+D-d21 in young mice (Fig. 6a). All these data indicate that transiently activated YAP/TAZ in ISMCs during intestinal wound healing seen in young mice may play an important role in the transformation of ISMCs into fibroblasts.

Similarly, in contrast to young mice, reduced activation of YAP/TAZ in ISMCs and reduced fibroblasts in ageing and advanced-age intestinal microenvironment were also observed in DSS-induced acute murine colitis (Supplementary Fig. 7b–e). To explore the relevance of YAP/TAZ activation seen in the murine colitis model, the human samples of three IBD patients were tested, and the activation of YAP/TAZ in ISMCs was also observed (Fig. 6h–k and Supplementary Fig. 7f). Thus, the activation of YAP/TAZ may be a common mechanism for the ISMCs responding to both mice and human intestinal inflammatory diseases.

### YAP/TAZ is required for ISMCs to transform into fibroblasts during intestinal wound healing

**Fig. 7:**
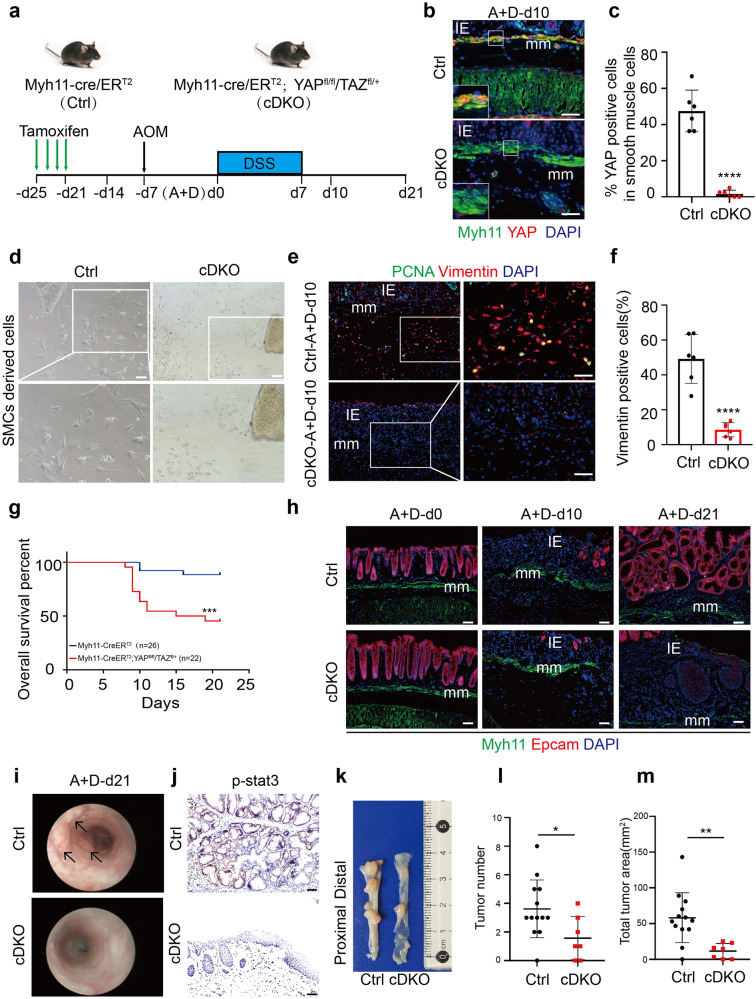
YAP/TAZ is required for ISMCs to transform into fibroblasts during intestinal wound healing. **a** Schematic overview of the tamoxifen induction to conditionally knockdown YAP/TAZ in ISMCs during the early phase of the CAC model. **b** Detection of Myh11 and YAP in ISMCs of muscularis mucosa at A+D-d10 in Myh11-cre/ER^T2^(Ctrl) and Myh11-cre/ER^T2^; YAP^fl/fl^/TAZ^fl/+^ (cDKO) mice. Scale bars, 50 μm. **c** The ratio of YAP-positive cells in muscularis mucosae at A+D-d10 in Ctrl and cDKO mice. *n* = 6, Data are mean ± SD. **d** SMCs-derived cells (SDCs) from the muscular layer of Ctrl and cDKO mice. Scale bars, 100 μm. **e** Detection of Vimentin and PCNA in the colon tissues of Ctrl and cDKO mice at A+D-d10. Scale bars, 50 μm. **f** The ratio of Vimentin^+^ fibroblasts in intestinal stromal microenvironment at A+D-d10 in Ctrl and cDKO mice. *n* = 6, mean ± SD. **g** Survival analysis of cDKO mice (red line; *n* = 22) compared to that of Ctrl mice (blue line; *n* = 26; *P* < 0.001 by log-rank test) during the early phase of the CAC model. **h** Immunofluorescence images of the temporal re-epithelialization process that occurs during the early phase of the CAC model in Ctrl and cDKO mice. Sections are immunolabeled for Epcam and Myh11. Scale bars, 50 μm. **i** High-resolution endoscopic images of the colon at A+D-d21 in Ctrl and cDKO mice. **j** Immunohistochemical staining of p-stat3 in colon tissues at A+D-d21 in Ctrl and cDKO mice. Scale bars, 50 μm. **k** Representative macroscopic images of CAC in Ctrl and survival cDKO mice at A+D-d63. **l** Tumor number per colon was measured at A+D-d63. Ctrl, *n* = 12; cDKO, *n* = 7, *P* < 0.05. Data are mean ± SD. **m** Tumor area per colon was measured at A+D-d63. Ctrl, *n* = 12; cDKO, *n* = 7, *P* < 0.01. Data are mean ± SD

To further demonstrate the role of activated YAP/TAZ in the transformation of ISMCs into fibroblasts during intestinal wound healing, we used Myh11-cre/ER^T2^; YAP^fl/fl^/TAZ^fl/+^ (cDKO) mice to conditionally knockdown YAP/TAZ only in SMCs (Fig. 7a). Two weeks after the tamoxifen induction, knockdown of YAP/TAZ in ISMCs did not affect normal tissue structure and resident fibroblasts on homeostasis (Supplementary Fig. 8a). However, the knockdown of YAP/TAZ in ISMCs led to significantly reduced fibroblasts derived from colonic muscle layer tissue in ex-vivo culture experiments. By nearly day 7 and day 14, we can observe proliferating fibroblasts-like cells around muscle layer tissue culture only from normal young mice, but not the non-fibroblasts-like cells observed from cDKO mice, which is also identified by Vimentin and PCNA IF staining (Fig. 7b–d and Supplementary Fig. 8e, f). Then we collected emigrated cells derived from smooth muscle (SDCs) of young and cDKO mice to coculture with colonic crypts. More colonic organoids were observed in crypts coculture with SDCs from young mice, compared to the same number of colonic crypts cultured alone or cocultured with SDCs from cDKO mice (Supplementary Fig. 8g, h). At the same time, we tried to activate YAP/TAZ in the ageing muscle layer during ex-vivo culture. Compared to untreated ageing-control, non-fibroblasts-like cells derived from the muscular layer were nearly not observed in the ageing muscular layer treated with YAP/TAZ activator (Supplementary Fig. 8i), which means recovery of YAP/TAZ in ageing smooth muscle may help to maintain ISMCs-fibroblasts transformation. Similar to the ex-vivo phenomenon, upon DSS-induced tissue damage in vivo, the knockdown of YAP/TAZ in ISMCs also led to significantly reduced fibroblasts in the intestinal microenvironment at A+D-d10 (Fig. 7e, f). Simultaneously, decreased re-epithelization during intestinal wound healing and higher mortality rates were observed in cDKO mice (Fig. 7g, h). Furthermore, decreased initiation of CAC in the rest of the survived cDKO mice was also observed (Fig. 7i–m and Supplementary Fig. 8b–d). All of these indicate that YAP/TAZ activation in ISMCs is required for their transformation into fibroblasts during intestinal wound healing, and conditional knockdown of YAP/TAZ in ISMCs leads to decreased tissue re-epithelization and simultaneously decreased CAC initiation.

## Discussion

**Fig. 8:**
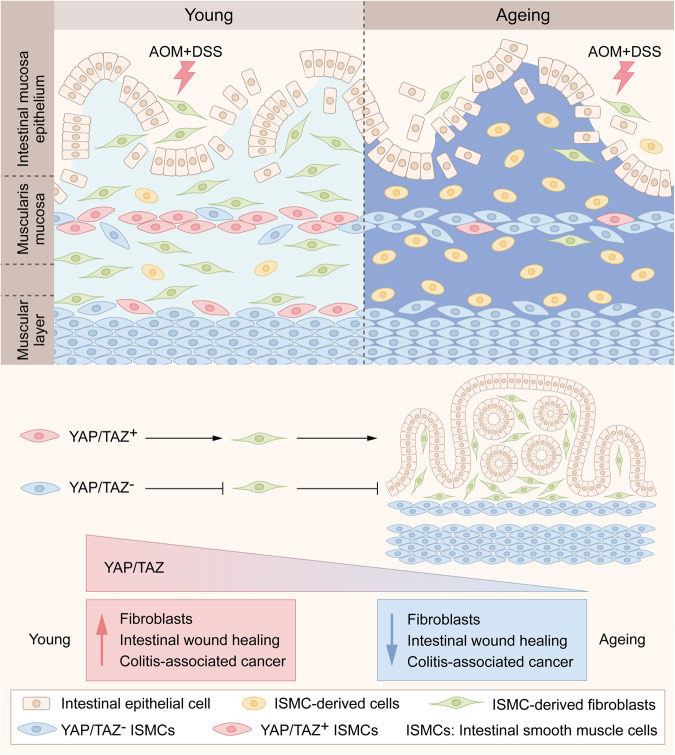
A schematic model of this study. Reduced YAP/TAZ-dependent transformation of intestinal smooth muscle cells (ISMCs) into fibroblasts potentially decreases intestinal wound healing and colitis-associated cancer (CAC) initiation in ageing mice

The incidence of the major types of cancer increases exponentially with ageing (middle-aged stage), but the age-related patterns of cancer incidence are not straightforward.^11^ For example, CRC shows a trend of increased incidence in the younger population,^1^ and the incidence of major types of cancer levels off or begins to decline at an advanced age, which means ageing may also play unfavorable roles in spontaneous cancer. While it is known that accumulated mutations with ageing, especially in epithelium stem cells, provide seeds for cancer formation,^31,32^ tissue microenvironment also plays important roles in tumor initiation and development.^33^ Here, we observed for the first time that reduced fibroblasts potentially transformed from ISMCs in ageing intestinal stromal microenvironment significantly decrease intestinal wound healing and simultaneously decrease initiation of murine CAC (Fig. 8), although ageing does not affect sporadic CRC under non-inflammatory conditions (Supplementary Fig. S3). These results indicate that although ageing could provide seeds (epithelial cells with accumulated mutations) for CRC, the soil (like reduced fibroblasts in our study) in the ageing intestinal stromal microenvironment may lose the ability to breed these seeds in some inflammation-driven CRC. This phenomenon is also likely to provide some insights into the trend of CRC in the younger population and the fact that the majority of the elderly population, after all, live without CRC.

Fibroblasts are important stromal cells in the tissue microenvironment, which orchestrate tissue repair,^4^ stem cell maintaining and self-renewal,^24,27–29^ and nearly all stages of cancer progression.^34,35^ Abundance of cancer-associated fibroblasts (CAFs) is associated with poor outcomes in cancer patients, especially in CRC.^36^ In our study, we observed that fibroblasts were significantly reduced in the ageing intestinal microenvironment during intestinal wound healing, accompanied by the dysregulated extracellular matrix, decreased tissue re-epithelization and decreased CAC initiation. The heterogeneity and origin of fibroblasts pose a big challenge for the treatment of IBD and CRC.^26,37,38^ Fibroblasts in CRC are generally considered to be derived from resident fibroblasts and stromal cells or from epithelial and endothelial cells via transdifferentiation.^25^Through lineage tracing, one study recently found that Lepr^+^ stromal cells are an important source of fibroblasts during colitis-associated cancer, but epithelial or bone marrow-derived α-SMA^+^ stromal cells do not play important roles in providing fibroblasts in CAC.^39^ Here, we identified a potential source of fibroblasts transformed from intestinal smooth muscle cells (ISMCs) orchestrating intestinal wound healing and CAC in young mice. Reduced transformation of ISMCs into fibroblasts could significantly decrease intestinal wound healing and CAC initiation in ageing mice.

In mammals, in response to tissue injury, the plasticity of tissue mature cells plays an important role in tissue regeneration in many organs, mainly including skin, intestine, lung, nervous system, liver, and pancreas.^40^ However, the plasticity of ISMCs upon intestinal tissue damage in colitis and CAC remains largely unclear before. Even though Joshua T. Mendell has ever used an unconditionally induced lineage-tracing system (Myh11-Cre/eGFP; mTmG) to observe the plasticity of ISMCs in a murine model of acute colitis, this unconditional tracing system could not strictly exclude the labeled cells from the resident Myh11^+^ Myofibroblasts or other cells derived from ISMCs before the colitis experiments.^41^ In our present study, we chose a conditional lineage-tracing system (Myh11-cre/ER^T2^; mTmG) for specifically tracing the newly transformed fibroblasts during intestinal wound healing instead and further confirmed this transformation during intestinal wound healing and found that these fibroblasts could also exist in the early phase of CAC. Of note, given the lack of a specific maker to identify smooth muscle cells and tissue-resident myofibroblasts, our lineage-tracing strategy could not be perfect. It is also difficult for us to perfectly exclude the possibility that remaining rare tissue-resident myh11^+^ myofibroblasts could be labeled at DSS-d7 and contribute to repopulating the fibroblasts during intestinal wound healing. But given the additional results in the ex-vivo system, we believe that the transformation of ISMCs into fibroblasts potentially plays a role in intestinal wound healing. Taken together, we believe our in vivo conditional lineage study and ex-vivo smooth muscle tissue study together move a solid step to explore smooth muscle cells in inflammatory bowel diseases.

Mechanistically, our work reveals that the activation of YAP/TAZ in ISMCs is required for the transformation of ISMCs into fibroblasts. YAP/TAZ are two transcriptional co-activators downstream of the Hippo pathway, which plays an important role in cell plasticity and cell fate determination during animal development and tissue regeneration.^20^ For example, the transient expression of exogenous YAP/TAZ in primary differentiated mouse cells can induce their conversion to a tissue-specific stem or progenitor cell state in the mammary gland, neuronal, and pancreatic exocrine cells.^42^ Upon tissue damage, YAP/TAZ-induced dedifferentiation or transdifferentiation of tissue mature cells was observed in mature colonic epithelium, hepatocytes, lung secretory cells, and basal epithelial cells of the skin, and the deletion of YAP/TAZ in these cells leads to the delayed wound healing and impaired tissue regeneration.^20,43–45^ The relationship between YAP/TAZ and smooth muscle cells is usually reported in vascular smooth muscle cells (VSMCs). YAP plays an important role in VSMC phenotypic switch process, especially in regulating VSMCs to fibroblast-like cells during vascular injury and neointima formation.^46,47^ However, the role of YAP/TAZ in the cell plasticity of ISMCs during intestinal wound healing still remains unknown. Here, we found the activation of YAP/TAZ in ISMCs during intestinal wound healing in mice for the first time. Our study further showed that the conditional knockdown of YAP/TAZ in ISMCs of young mice led to reduced fibroblasts in response to DSS-induced intestinal damage, which in turn decreased both intestinal wound healing and simultaneous CAC initiation. Altogether, this work not only reveals the plasticity of ISMCs during intestinal wound healing but also enriches the important role of YAP/TAZ in cell fate and plasticity in ISMCs.

With ageing, we found the transformation of ISMCs into fibroblasts during intestinal wound healing was significantly reduced, accompanied by decreased wound healing and decreased CAC initiation. Mechanistically, the reduction of YAP/TAZ activation in ISMCs during intestinal wound healing was observed in ageing (~14 months) and advanced-age (~25 months) mice. As a recent study shown by Sladitschek-Martens et al., declining YAP/TAZ activation was also observed in stromal cells during physiological ageing, orchestrating the ageing process.^48^ However, the mechanism underlying how ageing impairs the activation of YAP/TAZ in ISMCs and other stromal cells is still unclear, which is worthy of being explored in future studies. Besides, higher mortality associated with colitis was observed in the advanced-age group mice compared to that in the young and ageing mice. Given the discovery of the degeneration of intestinal stem cells in the small intestine and immune systems in advanced-age mice,^15,49^ we speculated that except the reduced fibroblasts transformed from ISMCs, the more severe degenerations in the immune system, colon stem cell, or other factors in advanced-age mice might lead to the higher mortality associated with colitis cooperatively. This phenomenon also reveals that although the ageing body is more suitable for cancer research, it is very important to consider the feasibility of experimental models in an ageing body. Besides, although the number of immune cells present after DSS-induced tissue damage seems similar between young and ageing mice in Supplementary Fig. S4, it is unwise to exclude them from the underlying mechanism in our study. For example, the function of young and ageing immune cells may be different during intestinal wound healing, which also needs more attention in the future study.

To establish the relevance of animal findings mainly observed in murine acute colitis and CAC models with humans, we further detected the activation of YAP/TAZ in ISMCs in clinical samples of IBD. Given the infeasibility of obtaining acute colitis samples of humans and the fact that only 15–20% of restrictedly diagnosed IBD patients are diagnosed after 60 years old,^50^ we only can confirm that the activation of YAP/TAZ in ISMCs exists in IBD patients. However, whether ageing leads to reduced expression of YAP/TAZ in ISMCs during intestinal wound healing in old people needs to be investigated in further clinical studies.

In conclusion, our work demonstrates that reduced fibroblasts in an ageing intestinal stromal microenvironment could impair intestinal wound healing and inflammation-driven CRC. Therefore, more attention should be focused on the change of intestinal stromal microenvironment with ageing to explore the relationship between ageing and inflammation-driven cancer. Additionally, our work also identifies a potential source of fibroblasts orchestrating intestinal wound healing and CAC initiation. Our novel findings may offer a new perspective to understand the mechanism of wound healing and inflammation-driven CRC in relation to the intestinal stromal microenvironment.

## Materials and methods

### Mice

Young (~3 months, ~25 g) male C57BL6/J mice were obtained from Charles River and bred until ~14 months and ~25 months as ageing and advanced-age mice, respectively. Only active ageing mice with a healthy weight (30–40 g) were enrolled in our study. Lgr5-EGFP-IRES-cre/ER^T2^; Myh11-cre/ER^T2^; Rosa26-mTmG; YAP-flox/TAZ-flox mice were obtained from the Jackson Laboratory and maintained on a C57BL6/J background. We crossed Myh11-cre/ER^T2^ and Rosa26-mTmG to obtain Myh11-cre/ER^T2^; Rosa26-mTmG mice for lineage-tracing experiments and then crossed Myh11-cre/ER^T2^ and YAP-flox/TAZ-flox mice to obtain Myh11-cre/ER^T2^; YAP^fl/fl^/TAZ^fl/+^ mice for conditional knockdown of YAP/TAZ in intestinal smooth muscle cells. All mice were housed under the same pathogen-free conditions in the animal facilities at West China Hospital (Chengdu, China) in compliance with animal care and relevant ethical regulation (20211485A).

### Murine sporadic colorectal cancer model

We administered AOM (10 mg per kg body weight, MilliporeSigma) intraperitoneal injection once a week for 6 weeks continuously in young (~3 months) and ageing (~14 months) mice. Through in vivo coloscopy system, we monitor the development of AOM-induced sporadic CRC dynamically at 15 and 25 weeks after the first injection of AOM. All the mice were sacrificed 25 weeks after the first injection of AOM. AOM-induced sporadic CRC usually occurs in the distal part of the colon and resembles the location of CRC at the distal colon and rectum in humans.

### DSS-induced murine experimental acute colitis

We used young (~3 months), ageing (~14 months) and advanced-age (~25 months) mice to establish a DSS-induced acute colitis model. We feed 2% Dextran Sodium Sulfate (DSS) (MP Biomedicals, Cat# 160110) in drinking water for 7 days, followed by normal water for 14 days. Continuous administration of DSS for up to 7 days (from DSS-d0 to DSS-d7) typically induces damage and ulceration in the colonic mucosa. The body weight during this model was measured dynamically. Details of this model and the scoring system for inflammation-associated histological changes in the colon have been described previously by Wirtz et al.^51^. (Score 0, none epithelial damage and infrequent inflammatory cell infiltration; Score 1–2, isolated focal epithelial and increased inflammatory cell infiltration; Score 3–4, mucosal erosions, ulcerations and submucosal presence of inflammatory cells; Score 5–6, extensive damage deep into the bowel wall and transmural cell infiltrations; Experimenters were blind during score data collection and analysis.)

### Murine colitis-associated cancer (CAC) model

We administered an intraperitoneal injection of AOM (10 mg per kg body weight) in young (~3 months) and ageing (~14 months) mice. One week later, we fed mice with 2% DSS (MP Biomedicals, Cat# 160110) in drinking water for 7 days, followed by normal water for 14 days, which is regarded as one cycle of colitis, including tissue repair and wound healing. After the first cycle of colitis, the precancerous lesions of CAC began to appear. To promote cancer development quickly, another two cycles of DSS-induced colitis were administered continuously. AOM/DSS-induced CRC usually occurs in the distal part of the colon. Details of this model have been described previously by Neufert et al.^19^.

### Human tissue samples

Three IBD tissue samples, including two ulcerative colitis samples and one Crohn’s disease sample, were collected at the West China Hospital (Chengdu, China). IBD tissue samples were fixed with 4% PFA for 24–48 hours at 4 °C. Then these tissues were dehydrated in 30% sucrose for 4 hours at 4 °C and subsequently embedded in OCT for frozen sections. The procedures of human tissue samples were approved by the Ethics Committee of the West China Hospital.

### Coloscopy and endoscopic colitis score

We used the in vivo coloscopy system (Mainz COLOVIEW® system, KARL STORZ) to monitor the development of DSS-induced colitis and AOM/DSS-induced CAC. Isoflurane (RWD Life Science, Cat# R510-22) was used for the anesthesia of mice. Endoscopic colitis score of the acute colitis model in young and ageing mice was blindly assessed using the murine endoscopic index of colitis severity (MEICS), which includes thickening of the colon, changes of the vascular pattern, fibrin visibility, granularity of the mucosal surface, and stool consistency.^52^

### Intestinal organoid

The colon was flushed gently, opened longitudinally, and then cut into 2 mm pieces. Gentle cell dissociation reagent (Stem cell, Cat# 07174, Canada) was used to isolate colonic crypts by incubating for 20 minutes at room temperature (15–25 °C). Crypt fractions were purified through successive centrifugation steps. Then the same volume of complete IntestiCult^TM^ organoid growth medium (Stemcell, Cat# 06005, Canada) and undiluted Matrigel (Corning, Cat# 356231) were added to culture intestinal organoid. Details of this model have been described previously by Sato T et al.^53^.

### Isolation and primary culture of colonic muscle layer tissue

The colonic muscular layer was isolated and cultured from young and ageing mice in homeostasis. Briefly, mice were sacrificed by cervical dislocation. The colon was removed, and the muscular layer was carefully peeled away with the help of the stereoscopic microscope. The muscular layer was thoroughly washed with sterile 5% penicillin-streptomycin in PBS for 30 minutes. After washing, we use sterilized scissors and tweezers to cut the muscle layer into 2 mm^2^ tissue pieces and culture tissue pieces into a 24-well plate. Then, 100 µl of DMEM medium containing 10% FBS and 5% penicillin-streptomycin was added to each well. After 3 hours, each well was supplemented with 2 ml of DMEM medium containing 10% FBS and 5% penicillin-streptomycin. For subsequent immunofluorescence staining, sterile 24-well plate slides were plated in 24-well plates before the tissue pieces were plated into 24-well plates. Usually, until nearly day 7, some fibroblast-like cells could be observed around some muscular layer tissues from young mice.

### Histochemistry and immunofluorescent staining

Colon tissues of mice were fixed with 4% PFA for 16–24 hours at 4 °C. The tissues were then dehydrated in 30% sucrose for 4 hours at 4 °C and subsequently embedded in optimum cutting temperature (OCT, Leica) compound and snap frozen. Then, 5-μm sections were prepared using a freezing microtome (Leica CM 1950). Cryosections were washed with PBS and incubated in a blocking buffer. Subsequently, the cryosections were incubated at 4 °C with the primary antibody overnight. For histochemistry staining, a DAB kit (Maixin, Fuzhou, China, Cat# DAB-0031) was used to detect the positive cells after incubation with the suitable secondary antibodies; then the cryosections were mounted with hematoxylin stain (Beyotime, China, Cat#C0105). For immunofluorescent staining, the cryosections were incubated with Alexa Fluor 488, Alexa Fluor 555, and Alexa Fluor 647 conjugated secondary antibodies (Thermo Fisher). The cryosections were mounted with a DAPI-containing medium (Beyotime, China, Cat# C1005). Fluroshield mounting medium (Abcam, Cat# ab104135) was used to preserve fluorescence when imaging tissues. Images were captured with the BX53 light microscope (OLYMPUS) and N-STORM&A1 confocal microscopes (Nikon). All of the antibodies used in this study are listed below. Antibodies were validated as protocol from the manufacturer’s website. Negative control and blank control were used to ensure the specificity of antibodies.

Primary antibodies used include: Rabbit antibody to Ki67 (clone SP6, Abcam, Cat# ab16667; RRID: AB_302459); Chicken antibody to GFP (Abcam, Cat# ab13970; RRID: AB_300798); Rabbit antibody to α-SMA (Abcam, Cat# ab5694; RRID: AB_2223021)；Mouse antibody to Myh11 (clone 1G12, Abcam, Cat# ab683; RRID: AB_2235569); Rabbit antibody to Epcam (Abcam, Cat# ab71916; RRID: AB_1603782); Mouse antibody to Stat3 (clone 124H6, Cell Signaling Technology, Cat# 9139; RRID: AB_331757); Rabbit antibody to p-Stat3 (clone D3A7, Cell Signaling Technology, Cat# 9145; RRID: AB_2491009); Rat antibody to CD45 (clone 30-F11, Cell Signaling Technology, Cat# 55307); Rabbit antibody to Laminin (Abcam, Cat# ab11575; RRID: AB_298179); Rabbit antibody to Collagen IV (Abcam, Cat# ab6586; RRID: AB_305584)；Rabbit antibody to Vimentin (clone D21H3, Cell Signaling Technology, Cat# 5741; RRID: AB_10695459); Mouse antibody to PCNA (Cell Signaling Technology, Cat# 2586; RRID: AB_2160343); Rabbit antibody to CD31 (Servicebio, Cat# GB11063-3); Rabbit antibody to Cleaved-Caspase3 (clone 5A1E, Cell Signaling Technology, Cat# 9664; RRID: AB_2070042); Rabbit antibody to YAP (clone D8H1X, Cell Signaling Technology, Cat# 14074; RRID: AB_2650491); Rabbit antibody to TAZ (Abcam, Cat#ab84927).

Secondary antibodies used included: Alexa Fluor 488 Goat anti-Rabbit IgG (H+L, Thermo Fisher Scientific, Cat# A-11034; RRID: AB_2576217); Alexa Fluor 555 Goat anti-Rabbit IgG (H+L, Thermo Fisher Scientific, Cat# A-21428; RRID: AB_141784); Alexa Fluor 488 Goat anti-Mouse IgG (H+L, Thermo Fisher Scientific, Cat# A-11001; RRID: AB_2534069); Alexa Fluor 568 Goat anti-Mouse IgG (H+L, Thermo Fisher Scientific, Cat# A-11004; RRID: AB_2534072); Alexa Fluor 488 Goat anti-Chicken IgY (H+L, Thermo Fisher Scientific, Cat# A-11039, RRID: AB_142924); Alexa Fluor 647 Goat anti-Rat IgG (H+L, Thermo Fisher Scientific, Cat# A-21247; RRID: AB_141778); Goat anti-rabbit IHC staining Kit (Zsbio, Cat# SP9001); Goat anti-mouse IHC staining Kit (Zsbio, Cat# SP9002);

### RNA-seq and analysis

Total RNA was extracted from colon tissues using Trizol reagent (Invitrogen, USA). The concentration of total RNA was quantified using the NanoDrop ND‐2000 (Thermo Scientific, MA, USA), and RNA integrity was assessed using the Agilent Bioanalyser 2100 (Agilent Technologies, Palo Alto, California, USA). Sample labeling, microarray hybridization, and washing were performed by the Shanghai OE Biotech Co., Ltd. (Shanghai, China). The threshold set for upregulated and downregulated genes was a fold change ≥ 2.0 and an unadjusted *P-*value ≤ 0.05. Three biological replicates were included at each time point in the microarray analysis.

### Flow cytometry

Isolation and analysis of intestinal lamina propria cells were performed using the standard procedure.^54^ The colon tissues were sectioned and digested in pre-digestive buffer (HBSS containing 3% FBS, 5 mM EDTA and 1 mM DTT) at 37 °C for 25 minutes. Then colon tissues were digested in Digestive buffer (RPMI 1640 containing 3% FBS; 0.2% type-IV collagenase, Thermo Fisher Scientific, Cat# 17104019; 3 mg/ml Dispase II, MilliporeSigma, Cat# D4693; 0.025% Nuclease, Sino Biological, Cat# SSNP01) at 37 °C twice for total 50 minutes. Isolated lamina propria mononuclear cells were collected and performed on flow cytometric analysis. The inflammatory cells in the colonic tissues were detected using primary antibodies (Biolegend) against CD45, CD3, CD4, CD8, NK1.1, CD19, CD11b, F4/80, GR-1, Ly6G, CD11c, MHC2, and CD103. FSC-A and FSC-H were used to identify single cells. The gating strategy includes: T cells (CD45-APC, CD3-APC/CY7, CD4-FITC, CD8-PE/CY7); B cells (CD45-APC, CD19-PE); Macrophages (CD45-PE, F4/80-FITC, CD11b-Percp); MDSC (CD45-APC/CY7, Gr-1-PE/CY7, CD11b-PE); Neutrophil (CD45-APC/CY7, CD11b-PE, Ly6G-APC); DC cells (CD45-APC/CY7, MHC2-APC, CD11c-FITC, CD103-PE, CD11b-PE/CY7); NK cells (CD45-APC/CY7, NK1.1-APC). The flow cytometric analysis was conducted using the NovoCyte flow cytometer (ACEA Biosciences), and the data were analyzed with the NovoExpress software (ACEA Biosciences).

Primary antibodies (All were purchased from Biolegend) used include: Anti-CD45-APC,clone 30-F11 (Cat# 103112; RRID: AB_312977); Anti-CD45-PE,clone 30-F11 (Cat# 103106; RRID: AB_312971); Anti-CD45-APC/CY7,clone 30-F11 (Cat# 103116; RRID: AB_312981); Anti-CD3-APC/CY7,clone 17A2 (Cat# 100222; RRID: AB_2242784); Anti-CD4-FITC,clone GK1.5 (Cat# 100406; RRID: AB_312691); Anti-CD8a-PE/CY7,clone 53-6.7 (Cat# 100721; RRID: AB_312760); CD19-PE (Cat# 115508; RRID: AB_313643); Anti-NK1.1-APC, clone PK136 (Cat# 108709; RRID: AB_313396); Anti-F4/80-FITC, clone BM8 (Cat# 123108; RRID: AB_893502); Anti-CD11b-PE/CY7, clone M1/70 (Cat# 101216; RRID: AB_312799); Anti-CD11b-PE, clone M1/70 (Cat# 101208; RRID: AB_312791); Anti-GR-1-PE/CY7, clone RB6-8C5 (Cat# 108416; RRID: AB_313381); Anti-Ly6G-APC, clone 1A8 (Cat# 127613; RRID: AB_1877163); Anti-CD103-PE, clone 2E7 (Cat# 121405; RRID: AB_535948); Anti-MHCII-APC, clone M5/114.15.2 (Cat# 107613; RRID: AB_313328); Anti-CD11c-FITC, clone N418 (Cat# 117305; RRID: AB_313774);

### Lineage tracing of intestinal smooth muscle cells (ISMCs)

For labeling ISMCs, young and ageing Myh11-cre/ER^T2^; Rosa26-mTmG mice were injected intraperitoneally with 3 mg tamoxifen (MilliporeSigma, Cat# T5648) dissolved in corn oil (Aladdin, Cat# C116023) per mouse once a day for two continuous days, starting from the sixth day of DSS feeding week in the first cycle of colitis in CAC model. GFP in ISMCs could be detected 36 hours after the beginning of tamoxifen induction.

### Conditional knockdown of YAP/TAZ in ISMCs

To conditionally knockdown YAP/TAZ in ISMCs, young Myh11-cre/ER^T2^ (Ctrl) and Myh11-cre/ER^T2^; YAP^fl/fl^/TAZ^fl/+^ mice was injected intraperitoneally with 2 mg tamoxifen (dissolved in corn oil) per mouse, once a day for four continuous days starting from 2 weeks before CAC model. We found inducible deletion of YAP/TAZ in Myh11-cre/ER^T2^; YAP^fl/fl^/TAZ^fl/fl^ mice cause rapid and lethal colonic pseudo-obstruction, but Myh11-cre/ER^T2^; YAP^fl/fl^/TAZ^fl/+^ mice own long-term surviving without impaired intestinal motility and other obvious unhealthy conditions after tamoxifen treatment (*n* > 10, data not shown), same as another study have reported.^55^

### Statistical analysis

Statistical significance between the two groups was determined using the two-tailed Student’s *t*-test. The Pearson correlation analysis was performed to determine the correlation between the groups. Statistical analysis was performed with GraphPad Prism8 software. For all statistical tests, the 0.05 level of confidence was accepted as a significant difference, * means *P* < 0.05, ** means *P* < 0.01, *** means *P* < 0.001, **** means *P* < 0.0001.

### Supplementary information

Supplementary Materials

## Data Availability

All data needed to evaluate the conclusions in this paper are present in the paper and/or the Supplementary Material. RNA-seq data have been submitted to the NCBI-GEO under the accession number GEO: GSE143693. Additional data related to this paper may be requested from the authors.
