# Supplementary material for: Reduced smooth muscle-fibroblasts transformation potentially decreases intestinal wound healing and colitis-associated cancer in ageing mice
Source: Signal Transduct Target Ther. 2023 Aug 9;8:294. doi: 10.1038/s41392-023-01554-w (PMC10409725; doi:10.1038/s41392-023-01554-w)
Supplement: Supplementary file 1 — Supplementary Materials [file 41392_2023_1554_MOESM1_ESM.docx]

Supplementary Materials for

**Reduced smooth muscle-fibroblasts transformation potentially decreases intestinal wound healing and colitis-associated cancer in ageing mice**

Yi Liu^1*^, Yanhong Ji^1*^, Ruiyi Jiang^1^, Chao Fang^2^, Gang Shi^1^, Lin Cheng^1^, Yinan Zuo^3^, Yixin Ye^1^, Xiaolan Su^1^, Junshu Li^1^, Huiling Wang^1^, Yuan Wang^1^, Yi Lin^1^, Lei Dai^1^, Shuang Zhang^4^, Hongxin Deng^1 #^

Correspondence to: [denghongx@scu.edu.cn](mailto:denghongx@scu.edu.cn)

**This PDF file includes:**

Figures. S1 to S8

Figure. S1.


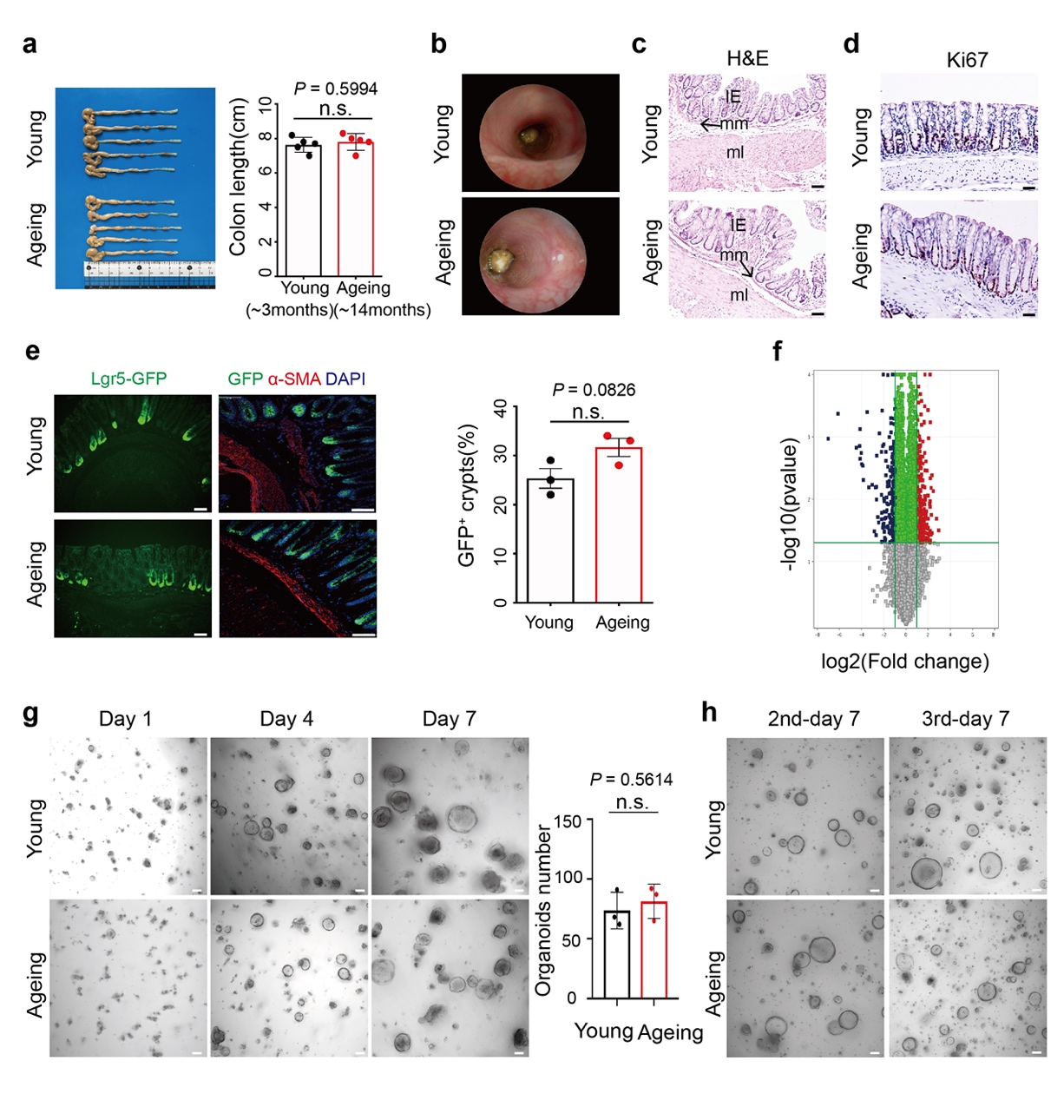


**Supplementary Fig. 1 Phenotypical characterization of homeostatic colon in young and ageing mice.** (**a**) The colon length of young and ageing mice in homeostasis. *n*=5, mean ± SD. (**b**) High resolution endoscopic images of young and ageing colon in homeostasis. (**c**) Representative images of H&E staining of young and ageing colon tissues in homeostasis. IE：intestinal mucosa epithelium, mm: muscularis mucosa, ml: muscular layer, these abbreviation apply to the whole study. Scale bars, 50 μm. (**d**) Immunohistochemical staining of Ki67 in homeostatic colon tissues of young and ageing mice. Scale bars, 50 μm. (**e**) Detection of Lgr5-GFP, GFP and α-SMA in colon tissues of young and ageing Lgr5-eGFP^+/-^ mice. The ratio of GFP^+^ crypts in colon tissues were measured. *n*=3, mean ± SD. Scale bars, 50 μm. (**f**) Volcano plot of RNA-seq data comparing whole transcripts of young and ageing colon tissues in homeostasis. 75 coding mRNA, 270 lncRNA and 45 cirRNA were differentially expressed by + 2-fold (*P* <0.05). Horizontal green line denotes *P*-value <0.05. Vertical green colored lines denote fold changes greater than ± 2 folds. *n*=3. (**g**) Intestinal organoids derived from 2000 crypts of young and ageing mice at day1，day4 and day7. Organoids number was measured at day7, *n*=3, mean ± SD. Scale bars, 100 μm. (**h**) Intestinal organoids derived from crypts of young and ageing mice at day7 of the second generation and third generation. Scale bars, 100 μm.

Figure. S2.


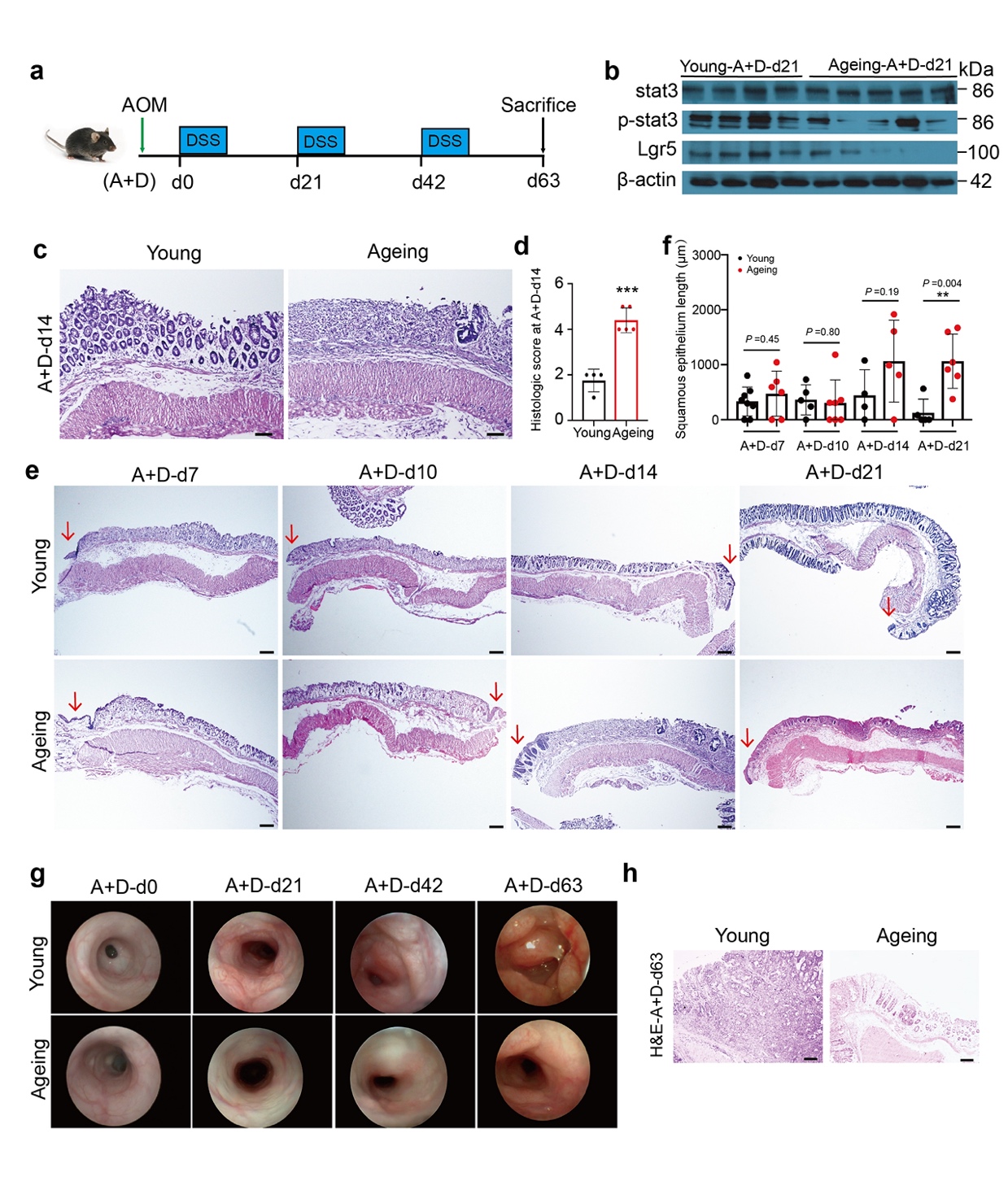


**Supplementary Fig. 2 Development of CAC in young and ageing mice, Related to Figure 2.** (**a**) Schematic overview of AOM/DSS-induced murine CAC model, A+D-d0 means normal mice. (**b**) Western blotting of the stat3, p-stat3, Lgr5 and β-actin proteins in colon tissues at A+D-d21 in young and ageing mice. Young, *n=*4; Ageing, *n*=5. (**c-d**) Representative H&E staining of colon tissues at 14 days after the beginning of DSS feeding. The histopathological grading of inflammation in young and ageing mice at A+D-d14 was recorded. Young, *n=*4; Ageing, *n*=5. Data are mean ± SD. Scale bars, 50 μm. (**e-f**) Representative H&E staining of colon tissues at 7, 10 ,14 and 21 days after the beginning of DSS feeding. Red arrow means the location near anal region. Scale bars, 200 μm. The length of squamous epithelium was recorded. Young-A+D-d7, *n*=8; Ageing-A+D-d7, *n*=6; Young-A+D-d10, *n*=5; Ageing-A+D-d10, *n*=7; Young-A+D-d14, *n*=4; Ageing-A+D-d14, *n*=5; Young-A+D-d21, *n*=5; Ageing-A+D-d21, *n*=6. Data are mean ± SD. (**g**) High-resolution endoscopic images of colon at 0, 21, 42 and 63 days during the CAC model in young and ageing mice. (**h**) H&E staining of young and ageing colon tissue at A+D-d63. Scale bars,100 μm.

Figure. S3.


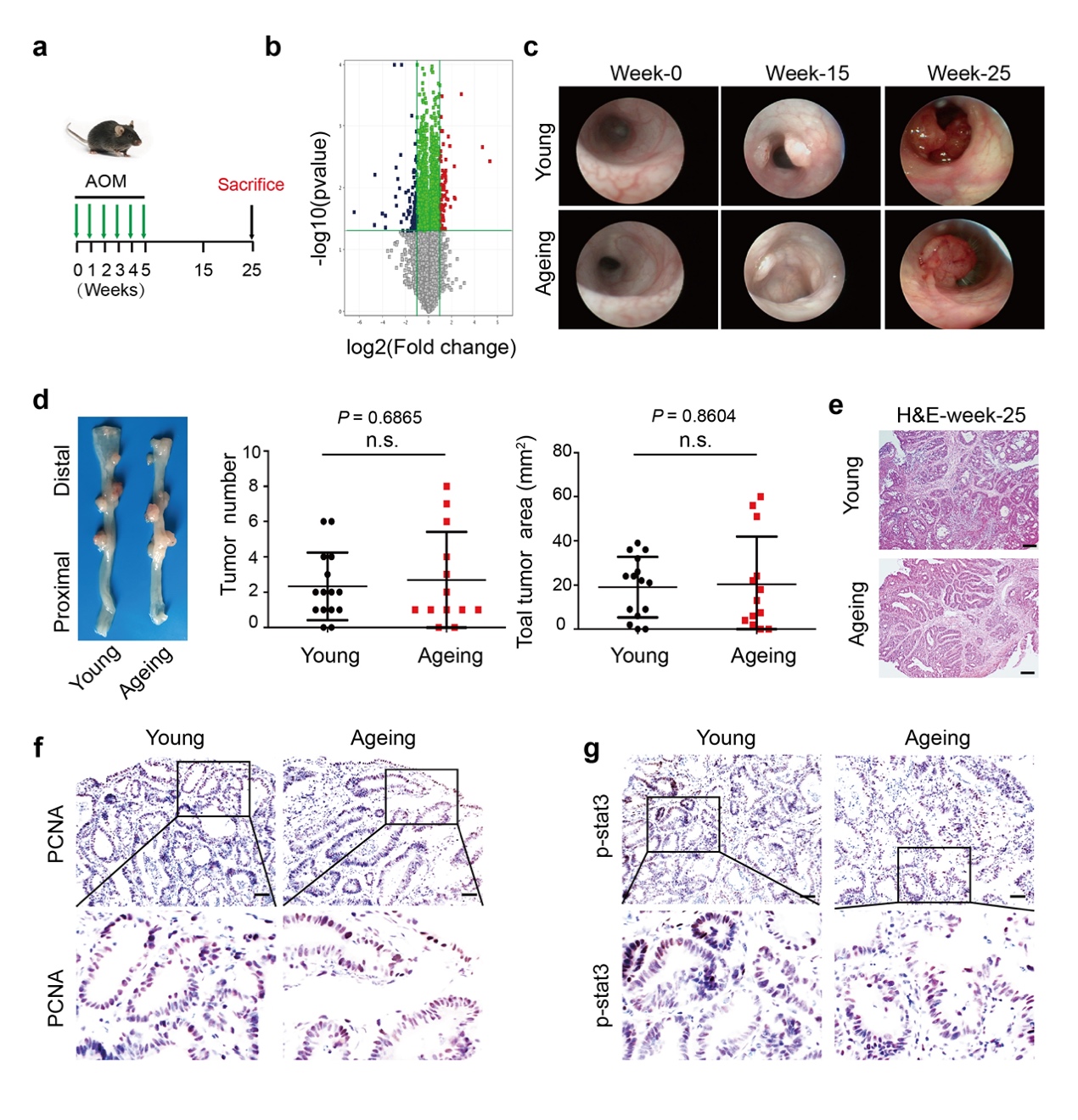


**Supplementary Fig. 3** **Development of multiple AOM-induced sporadic CRC in young and ageing mice****, Related to Figure 2.** (**a**) Schematic overview of murine model of multiple AOM-induced sporadic non-inflammatory CRC. (**b**) Volcano plot of gene expression data comparing whole transcripts of young and ageing colon tissues at week1. Only 86 coding mRNA, 68 lncRNA and 1 cirRNA were differentially expressed by + 2-fold (*P* <0.05). Horizontal green line denotes *P*-value <0.05. Vertical green colored lines denote fold changes greater than ± 2 folds. *n*=3. (**c**) High-resolution endoscopic images of young and ageing colon at 0, 15 and 25 weeks after the first AOM injection in the sporadic CRC. (**d**) Representative macroscopic images of sporadic CRC induced by multiple AOM injections at week 25 in young and ageing mice. Tumor number and tumor area per colon in young and ageing mice were measured. Young, *n*=15; Ageing, *n*=13; Data are mean ± SD. (**e**) H&E staining of young and ageing sporadic CRC at week 25. Scale bars, 100 μm. (**f**) Immunohistochemical staining of PCNA in colon tissues of young and ageing mice at week 25. Scale bars, 50 μm. (**g**) Immunohistochemical staining of p-stat3 in colon tissues of young and ageing mice at week 25. Scale bars, 50 μm.

Figure. S4.


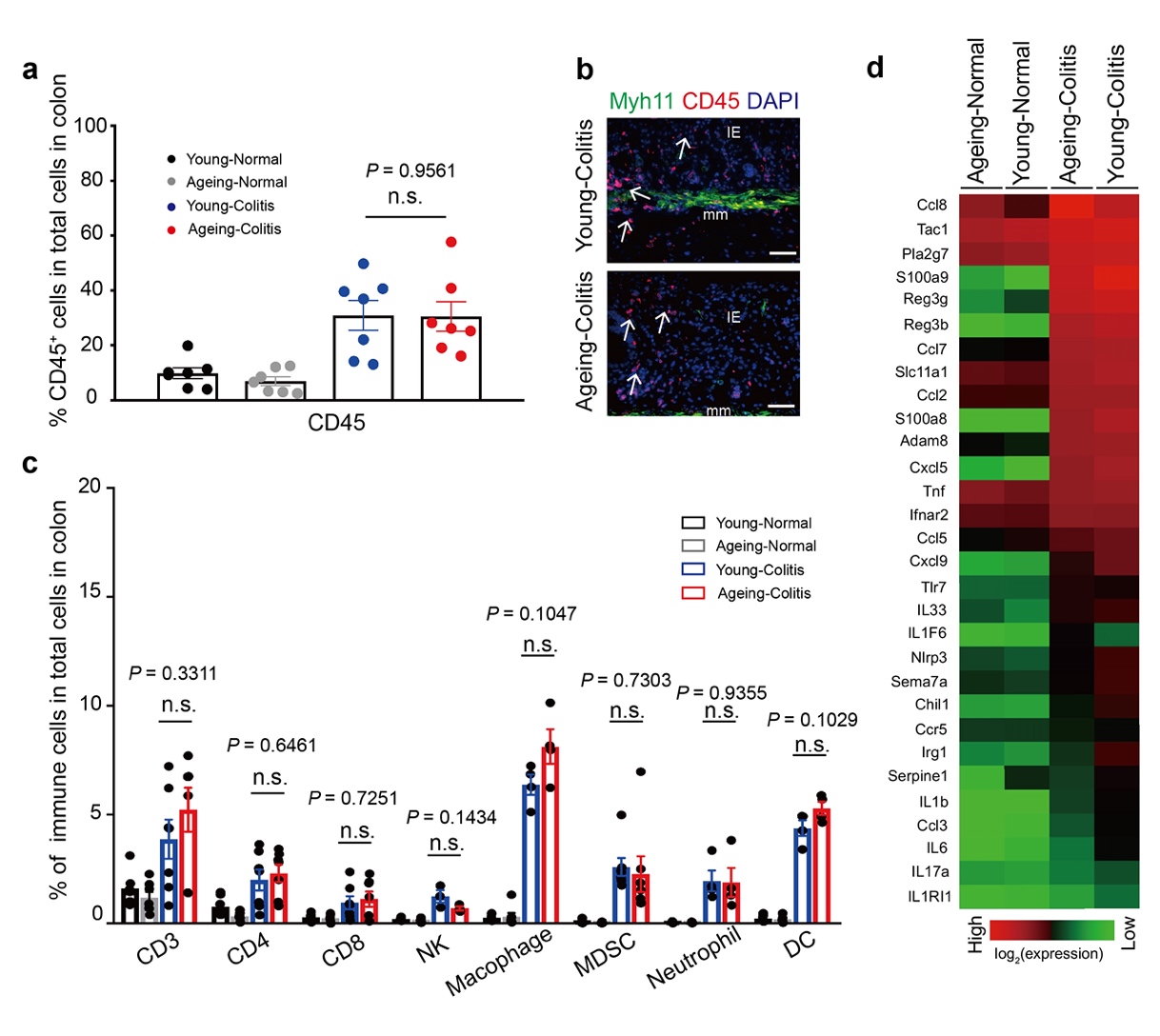


**Supplementary Fig. 4 Inflammatory characteristics during DSS-induced colitis in young and ageing mice.** (**a**) Flow cytometric analysis of CD45^+^ total immune cells in homeostatic colon and colitis colon in the early phase of CAC model in young and ageing mice. *n=*7 for each group, mean ± SD. (**b**) Detection of CD45 and Myh11 in colitis tissue in the early phase of CAC model at A+D-d10 in young and ageing mice. Scale bars, 50 μm. (**c**) Flow cytometry analysis of immune cells (CD3^+^ T cells, CD4^+^ T cells, CD8^+^ T cells, NK, Macrophages, MDSC, Neutrophil, and DCs) in homeostatic colon and colitis tissue in the early phase of CAC model in young and ageing mice. *n*=3-7/group, mean ± SD. (**d**) Heatmap of inflammatory factors expression from RNA-seq data of homeostatic colon and colitis colon in the early phase of CAC model at A+D-d10 in young and ageing mice. *n*=3 for each group.

Figure. S5.


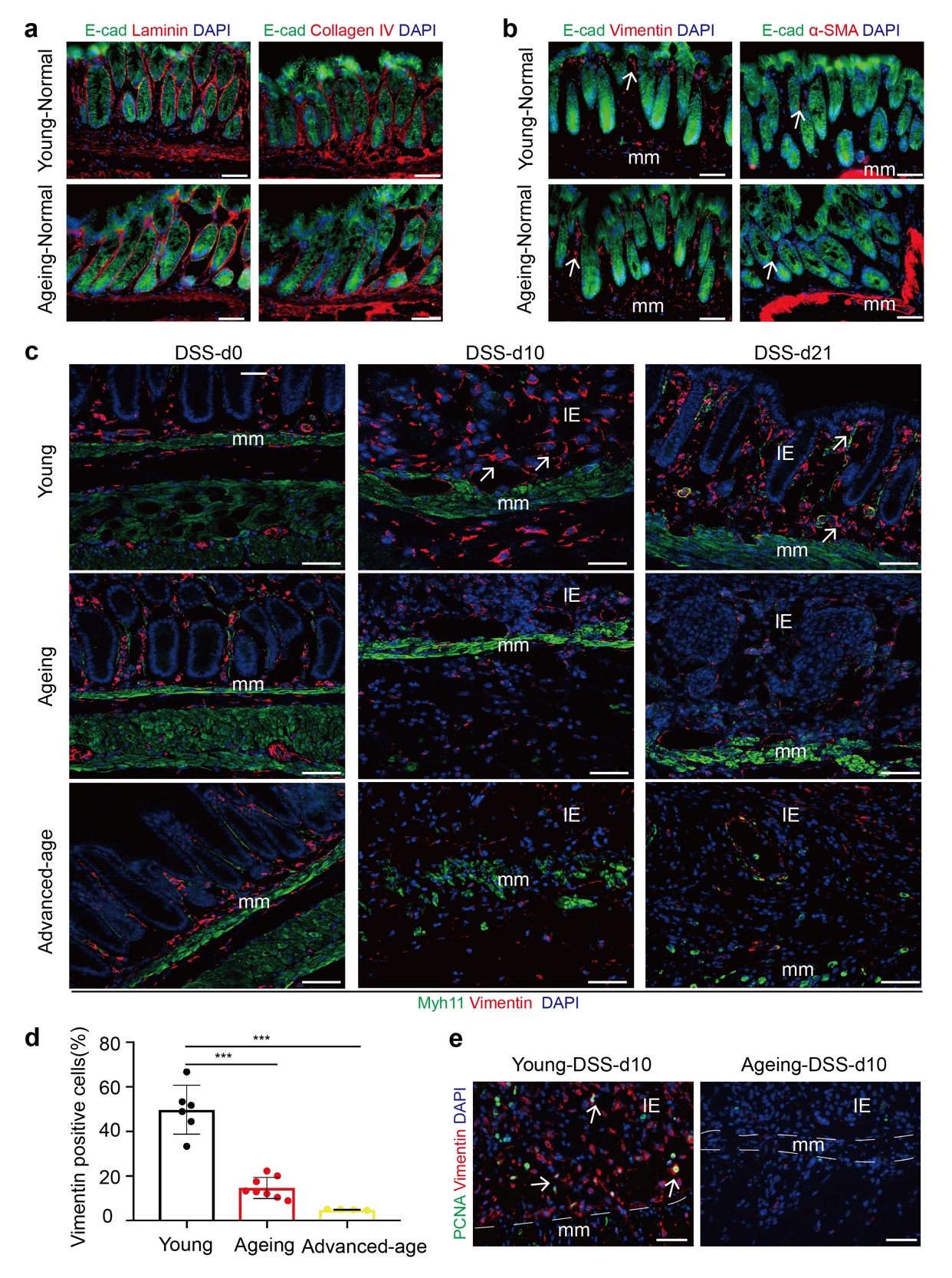


**Supplementary Fig. 5 Reduced fibroblasts with ageing during intestinal wound healing in DSS-induced colitis, Related to Figure 3.** (**a**) Detection of Laminin, E-cad and Collagen IV in homeostatic colon in young and ageing mice. Scale bars, 50 μm. (**b**) Detection of Vimentin, α-SMA and E-cad in homeostatic colon in young and ageing mice. Scale bars, 50 μm. (**c**) Detection of Vimentin and Myh11 at DSS-d0, DSS-d10 and DSS-d21 in young, ageing and advanced-age (~25 months) mice. Scale bars, 50 μm. (**d**) The ratio of Vimentin^+^ fibroblasts in the intestinal stromal microenvironment at DSS-d10 was recorded in young, ageing and advanced-age mice. Young, *n*=6; Ageing, *n*=8; Advanced-age, *n*=4. Data are mean ± SD. (**e**) Detection of Vimentin and PCNA in colon tissues at DSS-d10 in young and ageing mice. Scale bars, 50 μm.

Figure. S6.


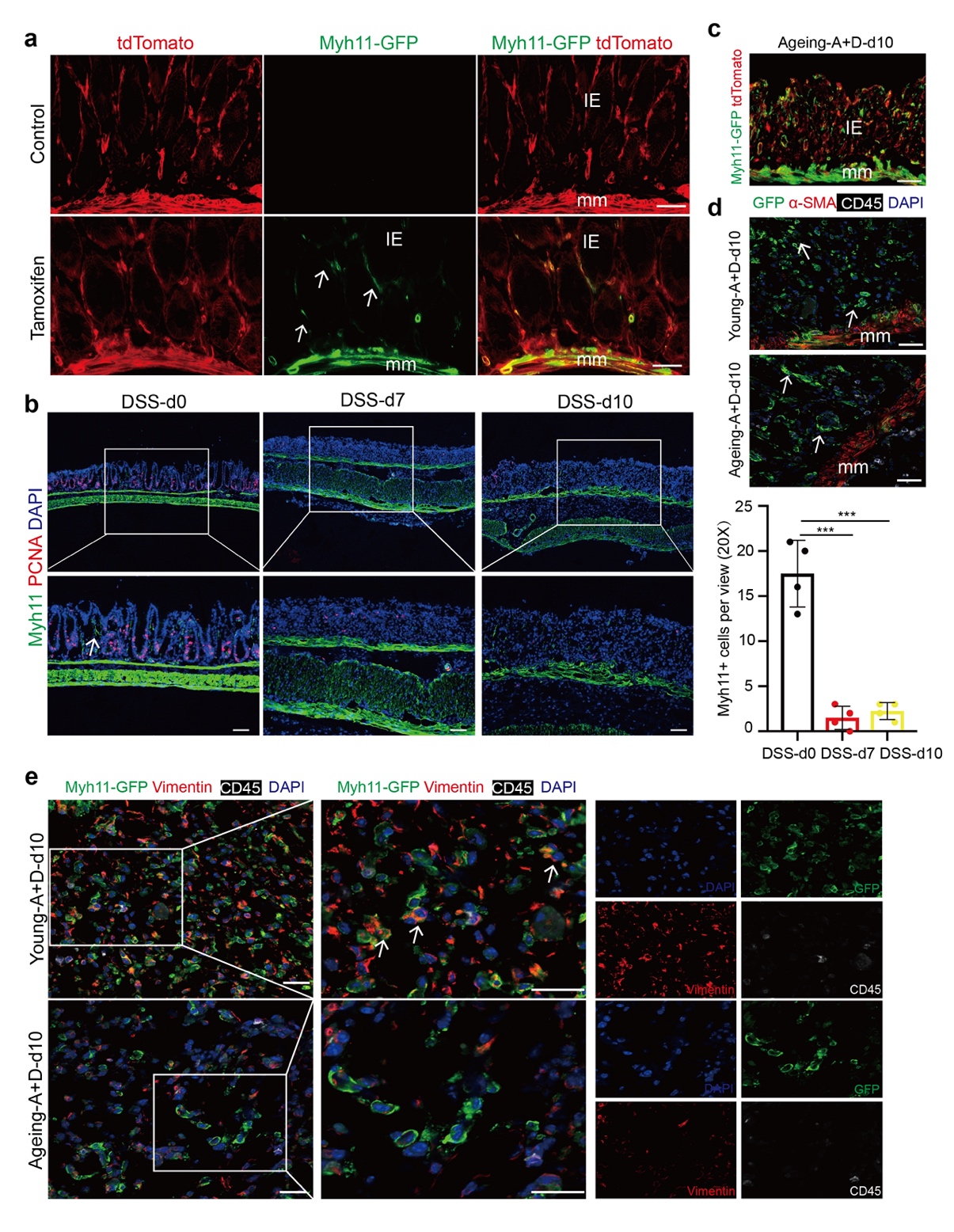


**Supplementary Fig. 6 Lineage tracing of ISMCs, Related to Figure 4.** (**a**) After two continuous days of tamoxifen (3 mg/day) induction starting from homeostatic condition, we detected the labeled cells derived from ISMCs (Green) and the total cells (tdTomato, red) in colon tissues. Scale bars, 50 μm. (**b**) Detection of PCNA and Myh11 in homeostasis or at DSS-d7 and DSS-d10 in young mice. Scale bars, 50 μm. The absolute number of Myh11^+^ cells per view (20X) was also detected at DSS-d0/7/10, *n*=4 for each group; Data are mean ± SD. (**c**) The labeled cells from ISMCs (Green) and the total cells (tdTomato, red) in colon tissues at A+D-d10 in ageing Myh11-cre/ER^T2^; Rosa26-mTmG mice. Scale bars, 50 μm. (**d**) Detection of the GFP, α-SMA and CD45 in colon tissues of young and ageing Myh11-cre/ER^T2^; Rosa26-mTmG mice at A+D-d10. Scale bars, 50 μm. (**e**) Detection of GFP, Vimentin and CD45 in intestinal mucosa epithelium at A+D-d10 in young and ageing Myh11-cre/ER^T2^; Rosa26-mTmG mice. Scale bars, 20 μm.

Figure. S7.


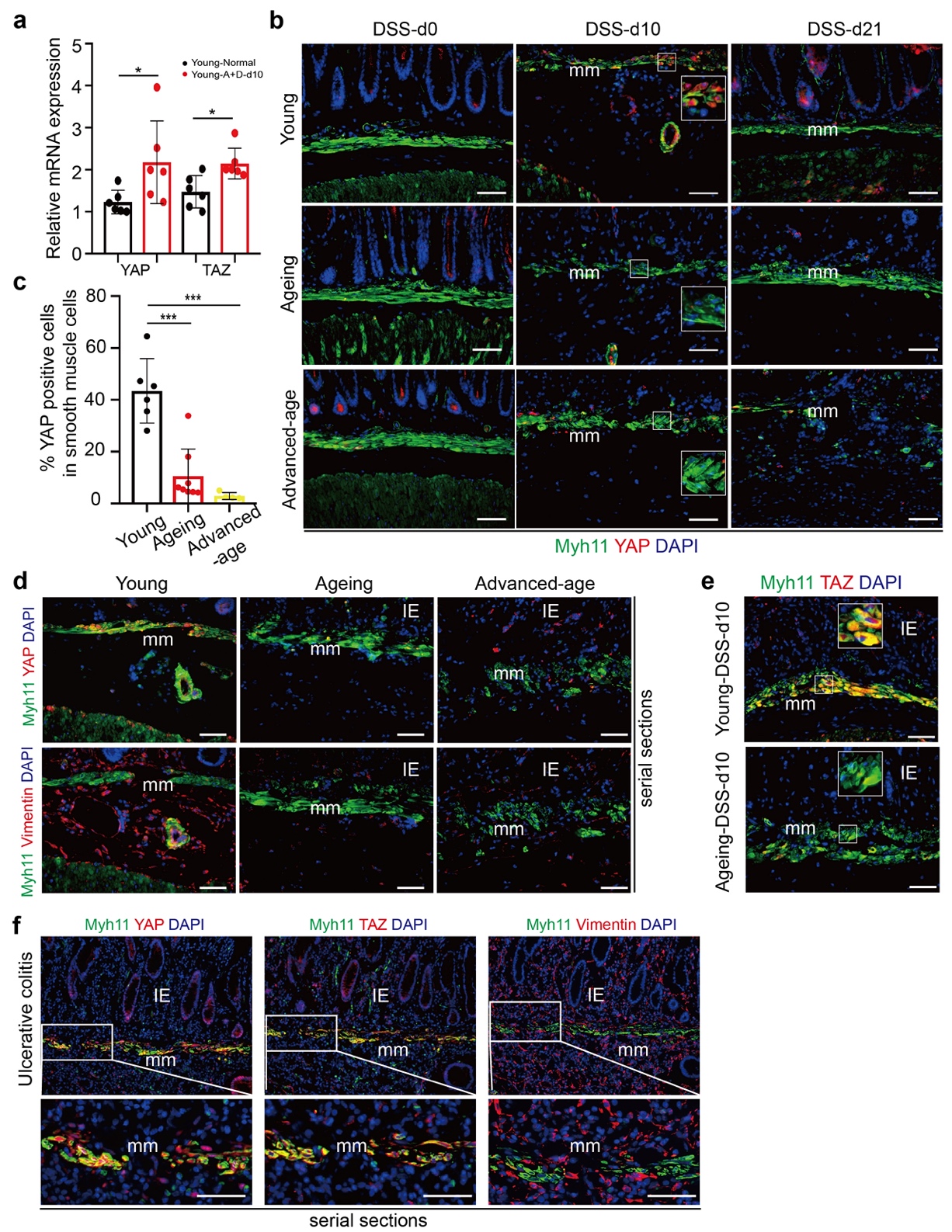


**Supplementary Fig. 7 The reduction of YAP/TAZ activation in ISMCs with reduced fibroblasts in acute colitis of ageing mice, Related to Figure 6.** (**a**) Relative mRNA expression of YAP and TAZ in the muscular layer of Young A+D-d10 mouse, compared to muscular layer of normal young mouse, n=6 for each group, Data are mean ± SD. (**b**) Detection of Myh11 and YAP in ISMCs of muscularis mucosa in homeostasis or at DSS-10 and DSS-21 in young (~3 months), ageing (~14 months) and advanced-age (~25 months) mice. Scale bars, 50 μm. (**c**) The ratio of YAP positive cells in ISMCs at DSS-d10 in young, ageing and advanced-age mice, Young, *n*=6; Ageing, *n*=8; Advanced-age, *n*=4. Data are mean ± SD. (**d**) Serial sections detection of YAP, Vimentin and Myh11 at DSS-d10 in young, ageing and advanced-age mice. Scale bars, 50 μm. (**e**) Detection of Myh11 and TAZ in ISMCs of muscularis mucosa at DSS-d10 in young and ageing mice. Scale bars, 50 μm. (**f**) Serial sections detection of YAP (red), TAZ (red), Vimentin (red) and Myh11 (green) in ulcerative colitis patients. Scale bars, 50 μm.

Figure. S8.


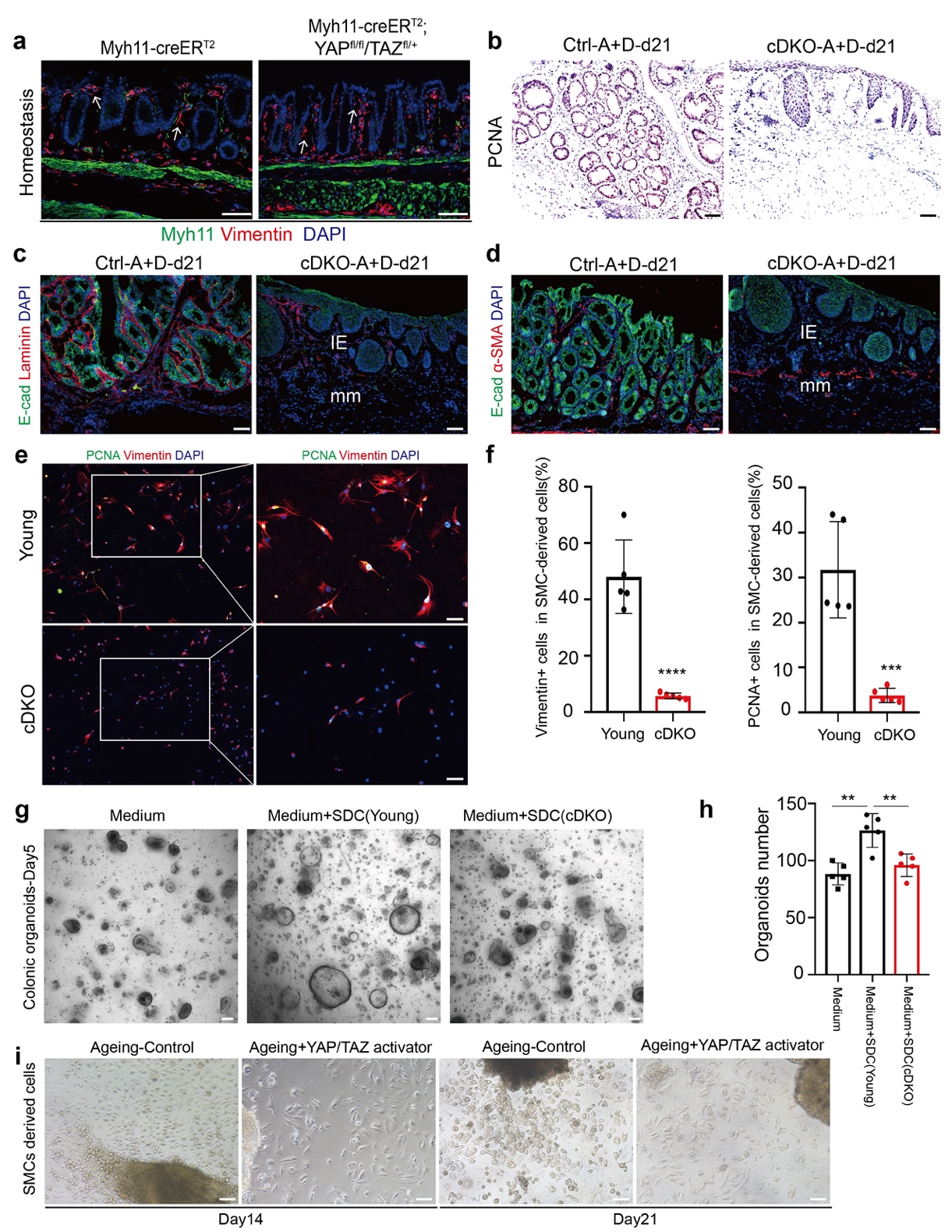


**Supplementary Fig. 8 Conditional knockdown of YAP/TAZ in ISMCs led to decreased CAC initiation, Related to Figure 7.** (**a**) Detection of Vimentin and Myh11 in Myh11-cre/ER^T2^ (Ctrl) mice and Myh11-cre/ER^T2^; YAP^fl/fl^/TAZ^fl/+^ (cDKO) mice in homeostasis. Scale bars, 50 μm. (**b**) Immunohistochemical staining of proliferation marker PCNA in colon tissues at A+D-d21 in Ctrl and cDKO mice. Scale bars, 50 μm. (**c**) Detection of E-cad and Laminin at A+D-d21 in colon of Ctrl and cDKO mice. Scale bars, 50 μm. (**d**) Detection of E-cad and α-SMA at A+D-d21 in colon of Ctrl and cDKO mice. Scale bars, 50 μm. (**e**) Detection of Vimentin, PCNA and DAPI of SDCs from muscular layer of young and cDKO mice. Scale bars, 50 μm. (**f**) The ratio of Vimentin^+^ cells and PCNA^+^ cells in SDCs from young and cDKO muscular layer. *n*=5 for each group, Data are mean ± SD. (**g-h**) Intestinal organoids derived from 400 crypts cultured alone or 400 crypts cocultured with 10000 SDCs from young or cDKO mice at day5, organoids number was measured, *n*=5 for each group, mean ± SD. Scale bars, 100 μm. (**i**) Compared to untreated ageing-control, YAP activator (PY60, 20μM, MedChemExpress) and TAZ activator (IBS008738, 20μM, MedChemExpress) were added at day 7 during ageing muscular layer culture, then we detect the smooth muscle derived cells at day14 and day21. Compared to untreated ageing-control, non-fibroblasts-like cells derived from muscular layer were not observed in ageing smooth muscle culture treated with YAP/TAZ activator. Scale bars, 50μm.
